# Multistage Self-Assembled Nanomaterials for Cancer Immunotherapy

**DOI:** 10.3390/molecules28237750

**Published:** 2023-11-24

**Authors:** Lamei Guo, Jinjun Yang, Hao Wang, Yu Yi

**Affiliations:** 1 Tianjin Key Laboratory of Hazardous Waste Safety Disposal and Recycling Technology, School of Environmental Science and Safety Engineering, Tianjin University of Technology, 391 Binshui Xidao, Xiqing District, Tianjin 300384, China; guolm2022@nanoctr.cn (L.G.); tjyjj_2014@tjut.edu.cn (J.Y.); 2 CAS Center for Excellence in Nanoscience, CAS Key Laboratory for Biological Effects of Nanomaterials and Nanosafety, National Center for Nanoscience and Technology (NCNST), No. 11 Beiyitiao, Zhongguancun, Beijing 100190, China; wanghao@nanoctr.cn

**Keywords:** cancer immunotherapy, in vivo self-assembly, drug delivery, multistage, stimuli–response, tumor microenvironment

## Abstract

Advances in nanotechnology have brought innovations to cancer therapy. Nanoparticle-based anticancer drugs have achieved great success from bench to bedside. However, insufficient therapy efficacy due to various physiological barriers in the body remains a key challenge. To overcome these biological barriers and improve the therapeutic efficacy of cancers, multistage self-assembled nanomaterials with advantages of stimuli-responsiveness, programmable delivery, and immune modulations provide great opportunities. In this review, we describe the typical biological barriers for nanomedicines, discuss the recent achievements of multistage self-assembled nanomaterials for stimuli-responsive drug delivery, highlighting the programmable delivery nanomaterials, in situ transformable self-assembled nanomaterials, and immune-reprogramming nanomaterials. Ultimately, we perspective the future opportunities and challenges of multistage self-assembled nanomaterials for cancer immunotherapy.

## 1. Introduction

Cancer immunotherapy is an advanced therapeutic strategy for cancers that boosts the body’s own immune system to fight cancer cells and has gradually changed the paradigm of cancer therapy in clinics [1,2,3]. In cancer patients, the tumor and its microenvironment usually suppress the host immune response by suppressing signaling pathways and metabolisms to escape the eliminations by immune cells such as T cells and natural killer (NK) cells [4,5,6]. Multiple defensive mechanisms, including defects in immune checkpoint expression, upregulations of immune-suppressive pathways, recruitments of immune-suppressive cells, etc., are involved in the immune surveillance of cancer cells. Different approaches have been developed for cancer immunotherapy, including immune checkpoint inhibitors, adoptive cell transfer, cancer vaccines, oncolytic viruses, lymphocyte-activating cytokines, etc. [7,8,9,10]. Currently, immune checkpoint inhibitors are one of the most successful therapeutics in cancer immunotherapy [11]. Since Ipilimumab, a monoclonal antibody that blocks cytotoxic T lymphocyte-associated protein 4 (CTLA-4), was first approved by the U.S. Food and Drug Administration (FDA) in 2011 [12], more than 20 immune checkpoint inhibitors have been marketed in the US, Asia, and Europe. Targets of these immune checkpoint inhibitors include CTLA-4, programmed cell death 1/programmed death ligand 1 (PD-1/PD-L1), and lymphocyte activation gene 3. Despite great successes, low responsive rates and immune-related adverse effects are still the main hurdles to cancer immunotherapy in the clinic [13,14].

Progresses in nanotechnology have promoted nanocarriers as a promising drug delivery approach for efficient cancer therapy [15,16,17,18,19,20,21,22,23] due to their advantages of targeted and controlled drug release in tumor sites, rational design in the size, structure, and morphology, spatiotemporal control in multi-functions, easy modification of biology-active moieties on the surface for recognizing tumoral biomarkers, reduction in side effects, flexibility to combine other synergistic therapies, etc. Various nanoformulations have been applied to improve the effectiveness of cancer, including polymeric nanoparticles [24,25,26,27,28], lipid-like nanoparticles [29,30,31,32,33,34], peptides and proteins [35,36,37,38,39], nucleic acids [40,41,42], dendrimers [43,44,45,46,47], inorganic nanomaterials [48,49,50,51,52,53,54,55,56], and biological membrane-based nanoparticles [57,58,59,60,61,62,63,64,65]. Up to now, significant successes have been made in the clinical translation of nanomedicines for cancer chemotherapy [66,67,68,69]. Several nanoparticle-based anticancer drugs have been marketed (Table 1) [70,71,72,73], including the liposomal formulations of doxorubicin (DOX) (e.g., Doxil), the albumin-bond paclitaxel (Abraxane), the polymeric micelles of paclitaxel (Genexol-PM), the small molecular micelles of paclitaxel (Paclical), etc. Recently, nanomedicines have also demonstrated promising potential to improve cancer immunotherapy. Some excellent reviews have highlighted these advances [74,75,76,77,78,79,80,81,82,83,84,85,86,87,88,89,90,91].

Nanomedicines are believed to accumulate in tumor regions via the hyperpermeable tumoral vasculature and dynamic leakage sites on the tumoral vessel wall, as well as the immature lymphatic drainage from tumor tissues, which is known as the enhanced permeability and retention (EPR) effect [92,93,94,95]. With a deeper understanding of the pharmacokinetics of nanomedicines and tumor microenvironments, nanomedicines have been developed from passive targeting systems to active targeting systems. However, due to the high heterogeneity of the EPR effect in the clinic [96,97,98] and various physiological barriers in human bodies, nanocarriers still encounter low delivery efficacy and poor therapeutic effect on tumors [99,100,101]. Thus, new strategies to overcome sophisticated physiological barriers are still urgent to improve the delivery efficacy of nanomedicines.

One strategy is to rational design and construct nanomaterials to deliver drugs in a multistage process to overcome multiple physiological barriers, thus achieving enhanced tumor accumulation and improved immunomodulation for cancer therapy [102,103,104,105]. These multistage nanomaterials can hierarchically change features, including size, morphology, surface modification, and core formation, upon external or internal stimuli, adapting to upcoming physiological environments like regional blood flow, tumor microenvironments, and intra/intercellular environments. Compared with conventional nanocarriers, these multistage nanomaterials are more likely accommodated to tumors in sophisticated physiological environments. In the following sections, we describe the typical biological barriers for nanomedicines, summarize the recent progress of multistage self-assembled nanomaterials for stimuli-responsive drug delivery, particularly in situ self-assembled nanomaterials and immune-reprograming nanomaterials, and perspective the future opportunities and challenges for multistage self-assembled nanomaterials for cancer immunotherapy.

## 2. Physiological Barriers for Nanomedicines

The pharmacokinetics of nanomedicines are strongly affected by administration routes and their physicochemical properties, including composition, size, shape, charge, surface modification, etc. [106,107]. Conventionally, an optimized nanocarrier for systemic drug delivery usually possesses several parameters [108,109,110,111], including (i) a proper size within 10–100 nm, (ii) a stealth shell such as a poly(ethylene glycol) (PEG) corona, (iii) tumor-targeting ligands on the surface, and (iv) a core for loading therapeutic cargos. Compared with small molecular drugs, these nano-scaled drugs present unique pharmacokinetics after systemic administration. They can circulate long in the blood flow and are more likely to escape from excreting through the kidney and being captured by the reticuloendothelial system (RES) in the liver, lung, and spleen, resulting in enhanced tumor accumulation. Some reviews have discussed the pharmacokinetics of nanoparticles after systemic administration in detail [112,113,114,115,116,117]. However, conventional nanomedicines still encounter the problems of low delivery efficacy and unsatisfied therapeutic outcomes due to the various physiological barriers in the body [118].

### 2.1. Physiological Barriers for Nanomedicines

**Figure 1:**
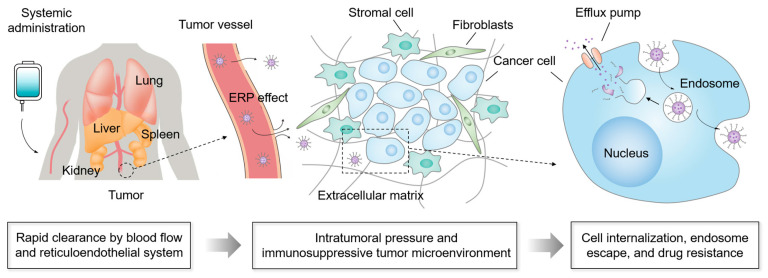
The biological barriers for nanocarriers for delivering drugs to tumors.

Upon systemic administration, nanomedicines encounter a series of sequential barberries before successfully arriving at the tumor sites (Figure 1) [119,120,121]. The rapid clearance in blood flow and uptake by the RES are the first obstacles to nanomedicines after administration, which, on average, contributes to more than 99% loss of injected nanomedicines [122,123]. The major challenges of nanomedicines during blood circulation are the degradation by enzymes, uptake by the RES and mononuclear phagocyte system, and excretion by the kidney. Of note, nanomedicines in the bloodstream face the problem of the formation of protein coronas due to the coverage by serum proteins [124,125,126,127], which inactivates the targeting ability of the ligand and facilitates the uptake by macrophages in the mononuclear phagocyte system, resulting in the non-specific accumulation and side effect to health organs such as liver, spleen, and lung. In addition, the blood flow also influences the stability of nanocarriers and usually causes burst release of the payloads. Another substantial barrier to nanocarriers is the high intratumoral pressure, which is associated with interrupted blood vessels, aggressive tumor cell proliferation, stroma cells, tumor-associated fibroblasts, and the extracellular matrix, impeding the convection of nanocarriers from tumoral vessels to tumor tissues and the deep penetration of nanocarriers within tumors [128,129,130]. Upon arrival at the tumor cells, cellular internalization and endosome escape are demonstrated as essential barriers for nanomedicines to approach therapeutic effects. Unfortunately, most nanomedicines possessing long blood circulation properties encounter the problem of poor uptake by the targeted cells, known as the “PEG dilemma” [131]. Meanwhile, nanomedicines installed with active-targeting ligands also face the risk of off-target effects caused by the formation of protein coronas [132,133]. In addition, drug resistance due to the drug efflux pumps has also proved to be a considerable obstacle for nanomedicines [134,135]. These biological barriers strongly hamper the clinical translation of nanomedicines from bench to bedside.

### 2.2. Passive and Active Targeting

The proposal of the EPR effect by Maeda in the 1980s established the base of nanomedicines [92]. Passive targeting nanocarriers refer to those that rely on the EPR effect to accumulate in tumors. These nanocarriers have been developed as the first generation of nano-scaled drug delivery systems. The equipment of stealth shells, such as PEGs and zwitterionic polymers [136], as well as the precise control of size in 10–100 nm, are two important features for designing this generation of nanocarriers. Some works have especially highlighted the advantages of sub-50 nm nanoparticles for deep penetration in thick fibrotic tumor models and metastatic tumor models [137,138,139,140]. Until now, passive targeting nanocarriers have achieved great success in clinical translations. Current marketed nanoparticle-based anticancer drugs all rely on passive targeting pathways to accumulate in tumors. However, evidence has pointed out that the liposomal DOX failed to show improvements in the objective response, overall survival, and progression-free survival rates via a meta-analysis in a total of 2589 patients in the clinic [141]. The unsatisfied performances of nanomedicines in the clinic are possibly due to the unspecific delivery and the highly heterogeneous EPR effect in patients [142,143,144,145]. Different patients, cancer types, and even different tumoral lesions in the same patient represent different responses to the EPR effect. To improve anticancer efficacy, ligand-installed nanocarriers for the active targeting of tumors have been developed as the second generation of nano-scale drug delivery systems. These active targeting nanocarriers rely on both the EPR effect to arrive at the tumor sites and the strong bind affinity to the specific biomarkers on targeted cancer cells and tumor vascular epithelial cells [146,147,148,149,150]. Up to now, different small molecules and biomolecules have been developed as targeting ligands [151,152,153], including folic acid [154,155,156], glucose [157,158,159,160], galactose [161], transferrin [162], antibodies [163,164,165], peptides [166,167], aptamers [168,169,170,171,172,173], etc. Notably, glucosylated nanocarriers have also been developed to cross the blood–brain barrier for drug delivery to the brain. For instance, Kataoka and coworkers reported a strategy for delivering glucosylated nanocarriers to the brain using glycemic control [174,175,176]. They conjugated the PD-L1 antibody with multiple PEG chains equipped with glucose via the C6 position, leaving the OH groups at positions C1, C3, and C4 to bind with the glucose transporter-1 overexpressed in brain capillaries. The PEG chains could detach in the tumor microenvironment to reinvigorate the potency of the antibody. In the orthotopic glioblastoma tumor-bearing mouse model, the glucosylated antibody achieved ~20-fold improvement in tumor accumulation compared with native antibody, resulting in potent antitumor immune response and immunological memory. Besides blood–brain barrier crossing, the same group also achieved the delivery of small interfering RNA (siRNA) to cancer stem cells (CSCs) using glucose-installed nanoparticles [177]. The glucose ligands on the sub-50 nm unimer polyion complex–assembled gold nanoparticle [178,179] specifically recognized the glucose transporter-1 overexpressed on the cell membranes of CSCs, resulting in a 2-fold higher delivery efficacy of siRNA to the orthotopic breast tumor model and 2.4-fold enhanced elimination of CSCs in tumor tissues compared with non-targeted nanoparticles. Up to now, at least 15 formulations of nanomedicines based on ligand-installed nanocarriers have been enrolled in clinical trials, including nine liposomal formulations (MM-302, C225-ILSDOX, anti-EGFR-IL-dox, SGT-53, SGT-94, Lipovaxin-MM, MCC-465, 2B3-101, and MBP-426), two bacterial-derived minicells (TargomiRs and EGFR(V)-EDV-Dox), two polymeric nanoparticles (BIND-014 and CALAA-01), one retroviral vector (Rexin-G), and one nanoparticle-based vaccine for smoking cessation (SEL-068). However, compared with the great success of antibody–drug conjugates in the clinic [180,181,182,183,184], the clinical translation of ligand-installed nanomedicines still encounters the hurdle of poor therapeutic outcomes in clinical trials. For instance, the BIND-014 was terminated in the phase II study due to its unsatisfactory therapeutic outcomes [185]. One reason is the heterogeneity of prostate-specific membrane antigen expression in each individual patient. To facilitate successful clinical translations of active targeting nanomedicines, it is important to establish proper and reliable models more closely to human tumoral environments and to develop non-invasion companion nano-diagnostic systems to monitor the therapeutic outcomes.

### 2.3. Cold Tumors and Hot Tumors

Besides the physiological barriers for nano delivery systems, the immunosuppressive microenvironment is also a critical hurdle for cancer immunotherapy, resulting in low response rates [186]. The immune checkpoint inhibitors-mediated antitumor response relies on the infiltration of T cells that recognize and kill tumor cells. However, immune checkpoint inhibitors are ineffective against cold tumors with little or no immune infiltration around cancer cells, leaving the immune system unable to attack and obliterate them effectively. This type of cancer is usually not sensitive to immunotherapy. In contrast, hot tumors have a large number of immune infiltrates around the cancer cells, and the cancer cells release signaling substances that attract immune cells and activate the immune response. This type of cancer is usually sensitive to immunotherapy. Current obstacles to treating cold tumors include the lack of effective antigens which provide targets for immunotherapy. In patients with cold tumors, there are few or no antigens on the surface of cancer cells, making it difficult for immune cells to identify and attack the cancer effectively. Therefore, it is necessary to further study the mechanism of tumor immune microenvironment in cold tumors to improve the outcomes of patients with cold tumors [187]. Tuning cold tumors into hot tumors is promising to improve the therapeutic effect of immune checkpoint inhibitors [188,189]. To this end, several strategies have been reported, such as promoting T cell priming and activation by increasing the antigen processing and presentation, enhancing T cell expansion by increasing the numbers of antigen-specific T cells, and augmenting T cell trafficking and infiltration by remodeling the tumor immune microenvironment, etc. [190]. Nanomedicines can contribute to these processes by targeting the cancer cells, tumor immune microenvironments, and peripheral immune system [191,192], providing assessments to overcome the barrier of tumor immunosuppressive microenvironment [193,194].

## 3. Stimuli-Responsive Nanomedicines for Cancer Immunotherapy

Increasing knowledge in tumor biophysics and biochemistry, especially tumor microenvironments, has promoted the development of stimuli-responsive nanocarriers for precise and specific drug release [195,196,197,198,199,200]. Triggered by internal stimuli such as tumoral acid [201,202,203,204,205,206,207,208], redox [209,210,211,212,213,214,215,216,217,218,219,220,221,222,223,224,225], hypoxia [226,227,228,229,230,231], and enzymes [232,233,234,235,236,237,238,239], or external stimuli like near-infrared (NIR) light and ultrasound [240,241,242,243,244,245,246,247,248,249,250], these nanocarriers are expected to specifically deliver and release drugs in the tumor site in a controlled manner.

### 3.1. Tumor Microenvironment-Responsive Nanomedicines

**Figure 2:**
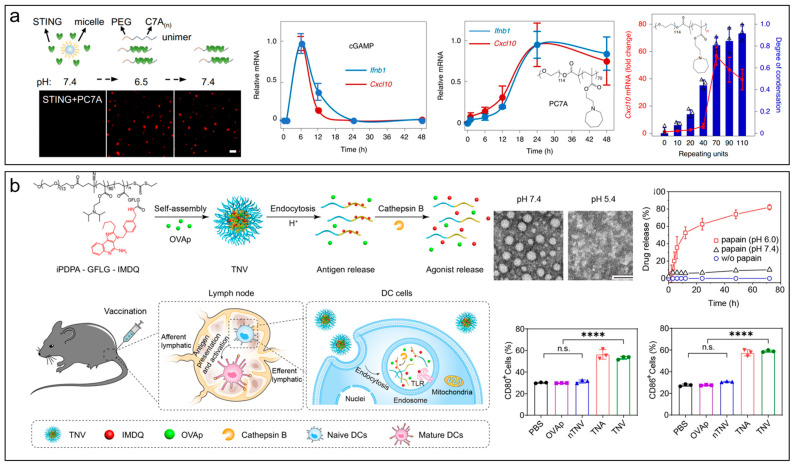
Tumor microenvironment-responsive nanomaterials for cancer immunotherapy. (**a**) PH-sensitive block copolymers formed biomolecular condensates with STING for prolonged cancer immunotherapy. Figure adapted with permission from Ref. [277] under the Creative Commons CC BY license. Copyright 2021, Springer Nature. (**b**) PH/enzyme-responsive nanoparticle for selective delivery of TLR agonists to active TLR7/8 receptor signaling at the endosomal membrane in dendritic cells. Data are shown as mean ± s.d., n.s.: not significant, **** *p* < 0.0001. Figure adapted with permission from Ref. [286]. Copyright 2022, American Chemical Society.

Tumor microenvironments refer to the surrounding environments in tumor regions, including the abnormal vasculature, acid, hypoxia, tumor-associated enzymes, redox, the extracellular matrix, stroma, intratumoral pressure, cancer stem cells, immune cells, etc. [251,252], which provide hotbeds for tumor proliferation, immune evasion, metastasis, and recurrence [253,254,255,256]. It has been recognized that the normalizing and remodeling of tumor microenvironments using antiangiogenic agents and antifibrosis drugs is a promising strategy to improve the therapeutic efficacy of cancers [257,258,259,260]. Advances in this topic are reviewed in detail elsewhere [260,261,262,263]. Additionally, stimuli-responsive nanomaterials targeting tumor microenvironments have attracted increasing attention in recent decades [264,265,266,267,268]. We summarized recent examples of stimuli-responsive nanosystems for cancer therapy in Table 2. These nanomaterials not only improve the efficacy of cancer therapeutics [269,270,271,272,273] but also provide opportunities for in situ monitoring of the levels and heterogeneities of contents in the tumor microenvironment [274,275]. For instance, Gao and coworkers reported a pH-responsive PEGylated polymer bearing a heptatomic ring with a tertiary amine (PC7A) that could simulate the stimulator of interferon genes (STING) pathway to enhance the cancer immunotherapy (Figure 2a) [276]. They showed that innate immunity was activated via the formation of STING-PC7A biomolecular condensates [277]. The polymer bound to a non-competitive pocket that differed from the natural STING ligand, resulting in a prolonged activation of the pathway. Besides the pathway activation, Liu et al. reported a pH-ultrasensitive transistor-like nanodetergent for selective cancer therapy via membranolysis [204]. This membranolytic block copolymer comprised a PEG shell, a pH-responsive segment with ionizable tertiary amines, and a hydrophobic segment. It achieved a >32-fold change in cytotoxicity with a 0.1 pH change. To monitor the tiny differences in endosome maturation pathway, Chen et al. engineered a library of pH-ultrasensitive polymeric nanophotosensitizer with a pH transition from 6.9 to 5.3. These nanophotosensitizers divided the endosome maturation into ten endocytic regions with a pH interval of 0.2, allowing the adjustment of pyroptosis-inducing activity by the targeted introduction of photodynamic oxidative stress into each region [278]. Besides pH-responsive polymers, pH-low insertion peptides (pHLIP) have also garnered much interest in cancer theranostics due to their unique ability to selectively target tumor acid and transform into transmembrane α-helix within tumor cell membranes [279,280,281,282,283]. For instance, Chu et al. reported a fusion protein of pHLIP and interleukin-2 (IL-2) for antitumor immunotherapy [284]. This protein was created by fusing the N-terminus of pHLIP with the C-terminus of IL-2, allowing for selective delivery to the acidic tumor microenvironment due to the low pH insertion property of pHLIP, thereby reducing the side effects. The presence of IL2 in tumor tissues promoted the proliferation of the CD8^+^ T and NK cells to suppress tumor growth, resulting in a 68% tumor inhibitory rate in a subcutaneous 4T1 tumor-bearing mouse model. In addition to tumor acid, Hu et al. reported a ROS-responsive delivery system for codelivery of anti-PD-L1 peptide and paclitaxel [285]. The peptides were modified on the nanoparticle’s surface, which could bind to the PD-L1 and induce its lysosomal degradation. The encapsulated chemodrugs were released under the overexpressed ROS in tumor cells for chemotherapy. This synergetic nanosystem promoted T cell infiltration and improved the anticancer potency for triple-negative breast cancers. Besides the single-responsive ones, multi-responsive nanosystems can further improve the specificity. For instance, Xia et al. reported a pH/enzyme-responsive nanoparticle for selective delivery of Toll-like receptor (TLR) agonists to active TLR7/8 receptor signaling at the endosomal membrane in dendritic cells (Figure 2b) [286]. They synthesized a pH-sensitive PEGylated polymer in which the TLR7/8 agonist imidazoquinoline was conjugated onto the side chain via a cathepsin B-cleavable GFLG peptide linkage. This nanosystem could release imidazoquinoline under the acidic environment and cathepsin B in the endosome and activate the TLR7/8 signaling, resulting in dendritic cell maturation and antigen presentation for immunotherapy.

### 3.2. External Stimuli-Responsive Nanomedicines

**Figure 3:**
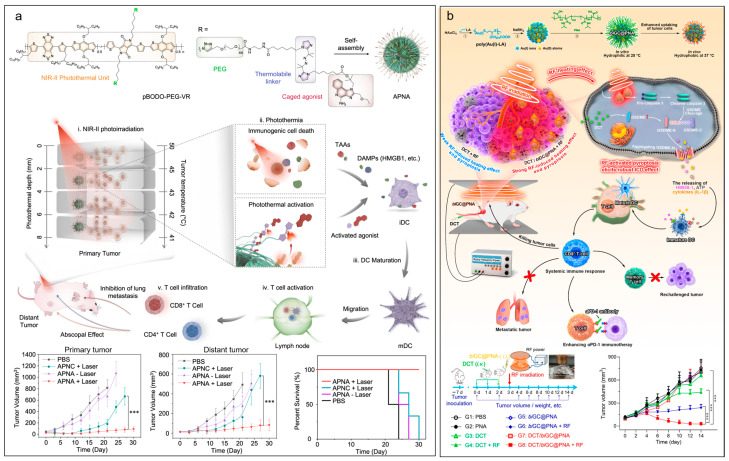
External stimuli-responsive nanomaterials for cancer immunotherapy. (**a**) NIR-II light activatable polymeric pronanoagonist for photothermal immunotherapy. Data are shown as mean ± s.d., *** *p* < 0.001. Figure adapted with permission from Ref. [328] under the Creative Commons CC BY license. Copyright 2021, Springer Nature. (**b**) Radiofrequency-activated bi-valent gold nanocluster for cancer immunotherapy. Data are shown as mean ± s.d., *** *p* < 0.001. Figure adapted with permission from Ref. [339]. Copyright 2023, American Chemical Society.

Triggering via external stimuli is also a promising strategy for approaching the site-specific delivery of therapeutic agents to tumors. The near-infrared (NIR) light (700–900 nm) is widely investigated in controlled drug delivery due to its relatively low scattering and tumor selectivity by local irradiation [324,325]. Currently, two NIR dyes, including the indocyanine green and the methylene blue, have been approved by the FDA for tumor diagnosis and image-guided surgeries. Compared with NIR light, NIR-II light (1000–1700 nm) has recently attracted increasing attention due to its reduced photon scattering and tissue autofluorescence [326,327]. For instance, Jiang et al. reported a NIR-II light activatable polymeric pronanoagonist for photothermal immunotherapy (Figure 3a) [328]. They construct the pronanoagonist by conjugating an immunostimulant onto an NIR-II semiconducting transducer using a thermo-responsive linker. Upon NIR-II irradiation, the photothermal effect of the pronanoagonist led to tumor ablation and immunogenic cell death, as well as the cleavage of a thermo-responsive linker to release the agonist for in situ immune activation in deep solid tumor (8 mm). Chen et al. developed a gold nano-adjuvant for the NIR-II light-triggered in situ tumor vaccine [329]. The nano-adjuvant comprised a multi-branched gold nanoparticle core with a localized surface plasmon resonance peak at 1032 nm and a shell containing hyaluronidases and CpG oligodeoxynucleotides. The hyaluronidases loosened the dense extracellular matrix of tumors by degrading the hyaluronic acids to make the nano-adjuvant penetrate the tumor tissue deeply, whereas CpG oligodeoxynucleotides bound the endosomal Toll-like receptor 9 to activate the antigen presentation cells. After penetrating deeply into the tumor, the nano-adjuvant induced the immunogenic cell death (ICD) effect under the irradiation of NIR-II light, thereby inhibiting tumor growth. Besides NIR and NIR-II lights, ultrasounds are also widely used in the diagnoses and treatments of many types of diseases in the clinic due to their advantages of deep tissue penetration, thermal effects, cavitation, and acoustic radiation forces [330,331]. Ultrasounds have been demonstrated to facilitate the release of drugs from liposomes, polymeric micelles, and micro/nanobubbles, improving the therapeutic efficacy [332,333,334,335]. However, the clinical translation of ultrasound-assisted nanomaterials is bumpy. A phase III clinical trial of ThermoDox, a thermosensitive liposomal DOX, combined with the high-intensity focused ultrasound for treating liver metastases tumors, did not meet the primary outcome. A post hoc analysis showed that ThermoDox was safe but invalid to increase the progression-free survival and the overall survival for the radiofrequency ablation [336]. An improvement in the overall survival was observed in a subgroup of 285 patients (41% of total) who underwent ultrasound treatments for 45 min or more, suggesting an opportunity for increasing efficacy. Combining immunotherapy provides new possibilities for ultrasound-assisted nanosystems. Li et al. reported a microbubble-assisted ultrasound-guided nanoplatform for cancer immunotherapy [337]. This platform was composed of a microbubbles core and a shell containing the spermine-modified dextran, 2′3′-cyclic guanosine monophosphate-adenosine monophosphate (cGAMP), and anti-CD11b antibodies. The decorated anti-CD11b antibodies enabled the nanocomplex to target antigen-presenting cells and efficiently deliver cGAMP under sonoporation, activating STING-mediated antitumor immunity. In another example, Jiang et al. reported a sono-activated semiconducting polymeric nanoagonist for immunotherapy of head and neck squamous cell carcinoma [338]. The sonodynamic semiconducting polymer was conjugated with a STING agonist MSA-2 via a singlet oxygen cleavable linker. The nanoagonist could generate singlet oxygen under ultrasound, resulting in the tumor cell death for triggering the ICD effect and the release of conjugated STING agonists for in situ activation of the STING pathway in synergy. Radiofrequency ablation is a commonly used thermal therapy in clinics. Zhang et al. recently reported a bi-valent gold nanocluster with a precise Au(I) ion/Au(0) ratio for cancer immunotherapy by inducing pyroptosis via radiofrequency (Figure 3b) [339]. The nanoclusters were synthesized by sequential reduction by a weak reducer lipoic acid and a strong reducer NaBH_4_ with the further modification of temperature-sensitive block poly(N-isopropylacrylamide-b-acrylic acid). Under radiofrequency, the nanoclusters induced pyroptosis of tumor cells and further elicited an ICD effect, resulting in an improved antitumor efficacy of αPD-1 immunotherapy to 4T1 tumor-bearing mouse model with synergy of decitabine.

## 4. Multistage Self-Assembled Nanomaterials for Cancer Immunotherapy

**Figure 4:**
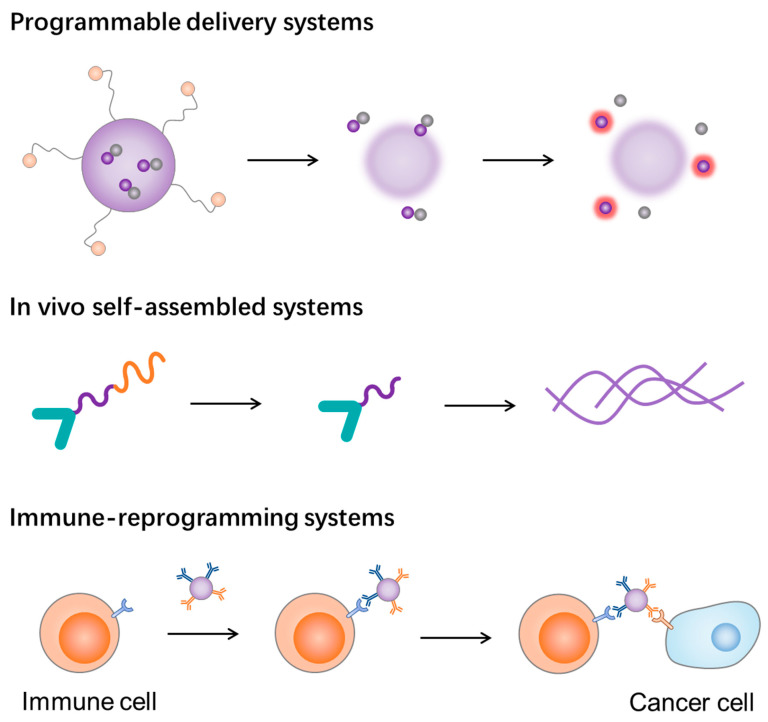
Strategies for construction of multistage self-assembled nanomaterials.

Compared with conventional active targeting nanocarriers that rely on the EPR effect and the ligand–receptor interaction, multistage self-assembled nanomaterials provide more chances to overcome the multiple biological barriers for cancer therapeutics. These barriers include but are not limited to the rapid clearance by the bloodstream and RES, off-target effect on tumors, intratumoral pressure, insufficient drug release, and immune-suppressive tumor microenvironment [340,341,342]. The advantages of multistage self-assembled nanomaterials include (1) they are flexible for changing the formulation to improve the ability to overcome the multiple biological barriers; (2) they can enhance the targeting efficiency and retention via programmable response or in situ self-assembly, thereby reducing the side effects; and (3) they can improve the immune response by modulating or reprogramming the tumor immune environment in a synergetic manner. An early example of multi-stage nanomaterials for cancer therapy was the mesoporous Si nanovector developed by Ferrari and coworkers [343,344,345,346,347,348,349]. These multistage nanocarriers were composed of nano-scaled pores and small therapeutic or diagnostic nanoparticles inside the pores, which could release out triggered by stimuli like acids. Recently, programmable nanomaterials that transform or self-assemble in situ have attracted increasing attention (Table 3) [350,351,352]. These nanomaterials enhance therapeutic efficacy by increasing the targeting affinity, penetrating ability, tumor retention, cell uptake, etc. In this section, we discuss the design and construction strategies for multistage self-assembled nanomaterials reported recently (Figure 4).

### 4.1. Programmable Delivery Systems

**Figure 5:**
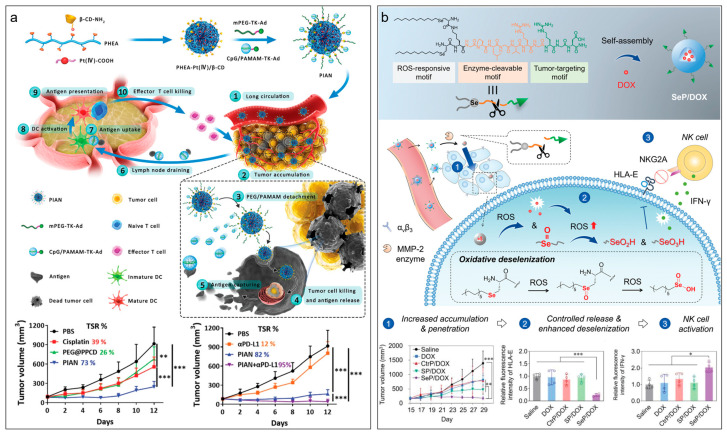
Multistage programmable delivery systems. (**a**) Programmable nanomedicine as in situ cancer vaccine for cancer immunotherapy. Data are shown as mean ± s.d., ** *p* < 0.01, *** *p* < 0.001. Figure adapted with permission from Ref. [384]. Copyright 2021, Wiley-VCH GmbH. (**b**) Selenopeptide nanoparticles improved the chemoimmunotherapy via the programmed delivery of DOX synergized with the NK cell-mediated immunotherapy. Data are shown as mean ± s.d., * *p* < 0.05, *** *p* < 0.001. Figure adapted with permission from Ref. [386]. Copyright 2022, Wiley-VCH GmbH.

To overcome the biological hurdles of nanomedicine, strategies such as PEGylation and crown detachment to increase blood circulation and tumor accumulation [364,365,366], installation of activable tumor-targeting ligands to increase tumor accumulation and cellular uptake [367,368,369], size-reduce to enhance tumor penetration [370,371,372,373,374,375,376,377], prodrug to reduce side effects [378,379,380,381,382,383], etc. have shown great potentials. One advantage of multistage programmable delivery systems is to integrate these individual strategies sequentially, corresponding to the sequential barriers after administration. For instance, Zhang et al. developed a programmable nanomedicine as an in situ cancer vaccine for cancer immunotherapy (Figure 5a) [384]. The nanomedicine had a core composed of poly-[(N-2-hydroxyethyl)-aspartamide]-Pt(IV)/β-cyclodextrin and a shell composed of CpG/polyamidoamine-thioketal-adamantane (CpG/PAMAM-TK-Ad) and PEG-thioketal-adamantane (PEG-TK-Ad). The CpG/PAMAM-TK-Ad and PEG-TK-Ad were attached to the core via host–guest interactions between β-cyclodextrin and adamantane. After administration, the PEG on the surface enabled long circulation in the blood, resulting in enhanced tumor accumulation. In tumor tissue, the high level of ROS triggered the detachments of PEG and CpG/PAMAM for improved cellular internalization of the core nanoparticles, which led to cell death and antigen release. The released antigen was further captured by the CpG/PAMAM, reached the tumor-draining lymph nodes, and internalized by dendritic cells. The activated dendritic cells presented antigens to T cells. The tumor antigen-specific effector T cells returned to tumor tissue to kill cancer cells. In a murine colorectal CT26 tumor model, this nanomedicine achieved a high growth-inhibitory activity of 73%. The further combination with anti-PD-L1 antibody resulted in a growth-inhibitory activity of 95%. Notably, 40% of the tumor-bearing mice were completely cured. In addition, the programmable nanosystem is also favorable for delivering combined drugs in different stages. For instance, Feng et al. reported an albumin nanoparticle for delivering PD-L1 inhibitor BMS-202 to the tumor microenvironment and cyclooxygenase-2 inhibitor celecoxib inside cells in a programmed manner [385]. The nanoparticle was composed of PEGylated human serum albumin derivatives that contained pH-responsive hexamethyleneimino groups, BMS-202, and celecoxib-poly(ethyleneimine) conjugates linked by reduction-responsive disulfide linkers. Via programmed delivery, the nanosystem achieved 3.1-fold in the infiltration of CD8^+^ T cells to tumors and almost complete inhiation in tumor growth in subcutaneous 4T1-bearing mice. In addition, to improve the tumor penetration and synergistic effect of the nanocarriers, Wei et al. developed a bioactive selenopeptide nanomedicine for enhanced tumor chemoimmunotherapy (Figure 5b) [386]. The selenopeptide was modularly designed with a tumor-targeting motif (RGD), a matrix metalloproteinase-2 (MMP-2) enzyme-cleavable linker (PLGVR), and a ROS-responsive seleno-amino acid tail. The selenopeptide was amphiphilic with a micelle structure in solution, which could encapsulate chemotherapeutics such as DOX. After systemic administration, the selenopeptide nanomedicine sequentially recognized α_v_β_3_ integrins on the tumor cell surface for improving tumor accumulation, reduced the size induced by MMP-2 enzyme for enhancing the tumor penetration, released DOX payload quickly in tumor cells under the high level of ROS, and activated the NK cells by the oxidative metabolites of selenopeptide for immunotherapy. Due to the programmable delivery and synergistic effect, the selenopeptide nanomedicine achieved a tumor growth inhibition efficacy of 86% in an orthotopic human breast MDA-MB-231 tumor-bearing mouse model, compared with 48% for DOX solely. To make more programmable biomaterials, introducing logic gates into nanomedicine design has attracted much attention [387,388,389]. For instance, Zhang et al. induced the concept of logic gates to construct a programmable polymer library [199]. Different stimuli-responsive units (e.g., light-, ROS-, glutathione-, acid-, esterase-, phosphates-responses) were integrated into these polymers with logic gates and hierarchical organizations, allowing to receive disease biomarkers as inputs and site-specifically release therapeutics (e.g., kinase inhibitors, drugs, and siRNA) as outputs.

### 4.2. In Vivo Self-Assembled Nanomaterials

**Figure 6:**
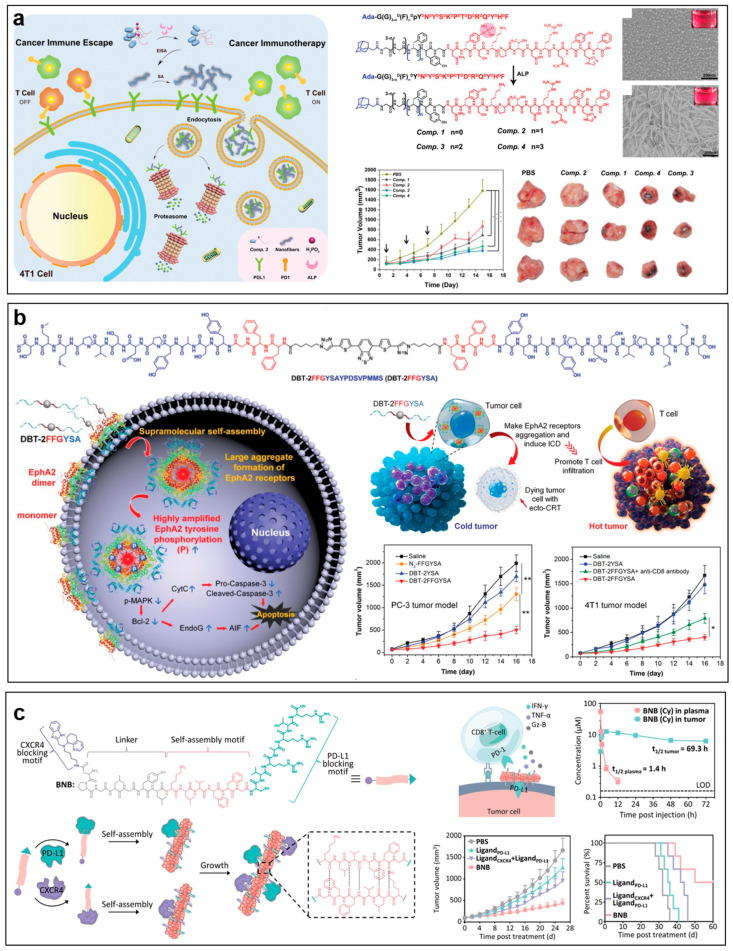
In vivo self-assembled nanomaterials for immunotherapy. (**a**) Degradation of PD-L1 in tumor cells by enzyme-instructed self-assembly. Data are shown as mean ± s.d., * *p* < 0.05, ** *p* < 0.01, *** *p* < 0.001. Figures adapted with permission from Ref. [393]. Copyright 2021, Wiley-VCH GmbH. (**b**) Self-assembled peptide system to facilitate aggregation of tumor-specific transmembrane receptors for converting cold tumors to hot ones. Data are shown as mean ± s.d., * *p* < 0.05, ** *p* < 0.01. Figures adapted with permission from Ref. [394]. Copyright 2021, Wiley-VCH GmbH. (**c**) In vivo self-assembled bispecific nano-blocker for improving tumor immunotherapy. Figures adapted with permission from Ref. [395]. Copyright 2023, Wiley-VCH GmbH.

In vivo self-assembled nanomaterials can transform or self-assemble in tumor tissues in situ triggered by internal or external stimuli. The self-assembly process is governed by both thermodynamics and kinetics, which provide different assembled structures and new biological functions. Via the rational design of building blocks and control of thermodynamics and kinetics, in vivo self-assembled nanomaterials have advantages such as enhanced tumor accumulation and retention, improved tumor penetration, increased cellular internalization, etc., enabling improved immunotherapy outcomes. For instance, by tuning the self-assembly properties and kinetics of peptide building blocks, either retention in the cell membrane or rapid cell entry was achieved, resulting in different biological activities [390,391]. To overcome the vaccine’s hurdles on poor lymph node delivery and dendritic cell uptake, Wang et al. reported an in situ phase transitional polymeric vaccine [392]. The vaccine was composed of a thermoresponsive poly(N-isopropylacrylamide) backbone modified with photothermal conversion cyanine and antigen peptide OVA_257–264_ (peptide sequence: SIINFEKL) on the side chains. The low critical solution temperature of the polymer was tuned to be 40 °C. During lymph node draining, the polymers retained a small size of 24 nm. Upon arrival at the lymph node, they transformed into 483 nm-sized particles triggered by laser, resulting in improved endocytosis by lymph node-resident dendritic cells. This laser-induced dynamic size modulation strategy induced a rapid and robust immune response in the subcutaneous B16-F10-OVA melanoma tumor-bearing mouse model. In addition to cancer vaccines, in vivo self-assembled systems have also shown promising potential to improve therapeutic efficacy. For instance, Wang et al. reported an enzyme-instructed self-assembly (EISA) peptide to selectively degrade PD-L1 in cancer cells for improved cancer therapy (Figure 6a) [393]. The peptide included a PD-L1-targeting motif, an alkaline phosphatase (ALP)-responsive phosphorylated tyrosine, and a self-assembly module containing phenylalanines and an adamantine (peptide sequence: Ada-GG^D^F^D^F^D^N^D^Y^D^S^D^K^D^P^D^T^D^D^D^R^D^Q^D^Y^D^H^D^F). After dephosphorylation by ALP and binding to PD-L1 on tumor cell membranes, the peptide self-assembled into nanofibers around PD-L1 in situ. The resulting peptide self-assemblies and PD-L1 were further internalized and degraded by the proteasome pathway in the cytoplasm. Interestingly, the in situ self-assembly happened in ALP-overexpressed murine breast cancer 4T1 cells instead of in normal human liver LO2 cells expressing low levels of ALP. Due to the selective degradation of PD-L1, the self-assembled peptide resulted in a tumor volume decrease of 23.7% in subcutaneous 4T1-bearing mice. In addition, the in situ self-assembled peptide can also induce the aggregation of receptors to activate anticancer signaling pathways. For instance, Li et al. reported an in situ self-assembled peptide system to facilitate the aggregation of tumor-specific transmembrane Eph receptor A2 (EphA2) for converting cold tumors to hot ones [394] (Figure 6b). The peptide concluded a central fluorophore 4,7-di(thiophen-2-yl)-2,1,3-benzothiadiazole and two peripheral EphA2-targeted self-assembled peptides (peptide sequence: FFGYSAYPDSVPMMS). The peptide bond specifically to EphA2 promoted cancer malignancy and induced the aggregation of the receptors, resulting in the activation of the antitumor pathway and visualization of EphA2 receptors in a fluorescence turn-on manner. By inducing immunogenic death and recruiting massive tumor-infiltrating T cells, the peptide also efficiently converted immunologically cold tumors to hot ones. To further overcome the hurdles of poor infiltration of T cells and tumor penetration of antibodies induced by ECM, Hu et al. reported an in situ self-assembled bispecific peptide that targeted both C-X-C chemokine receptor type 4 (CXCR4) and PD-L1 (peptide sequence: AMD070-DPGLGYLKLVFFGCVRARTR) [395] (Figure 6c). The rapid formation of CXCR4/PD-L1-targeted nanoclusters on tumor cell surfaces in situ could enhance the blockages of both CXCR4 and PD-L1, resulting in reductions in ECM component accumulation and solid tumor stress (to 44%). By improving T cell activation and infiltration, this in situ bispecific self-assembled system achieved a tumor growth inhibition efficacy of 74% compared with 24% for PD-L1 in the subcutaneous mouse urothelial carcinoma MB49 tumor-bearing mouse model. Of note, compared with antibodies, this nanosystem had the advantages of rapid blood clearance (elimination half-life (t_1/2β_) = 1.4 h) and prolonged tumor retention (t_1/2β_ = 69.3 h), providing possibilities to overcome the potential systemic side effect.

### 4.3. Immune-Reprogramming Nanomaterials

**Figure 7:**
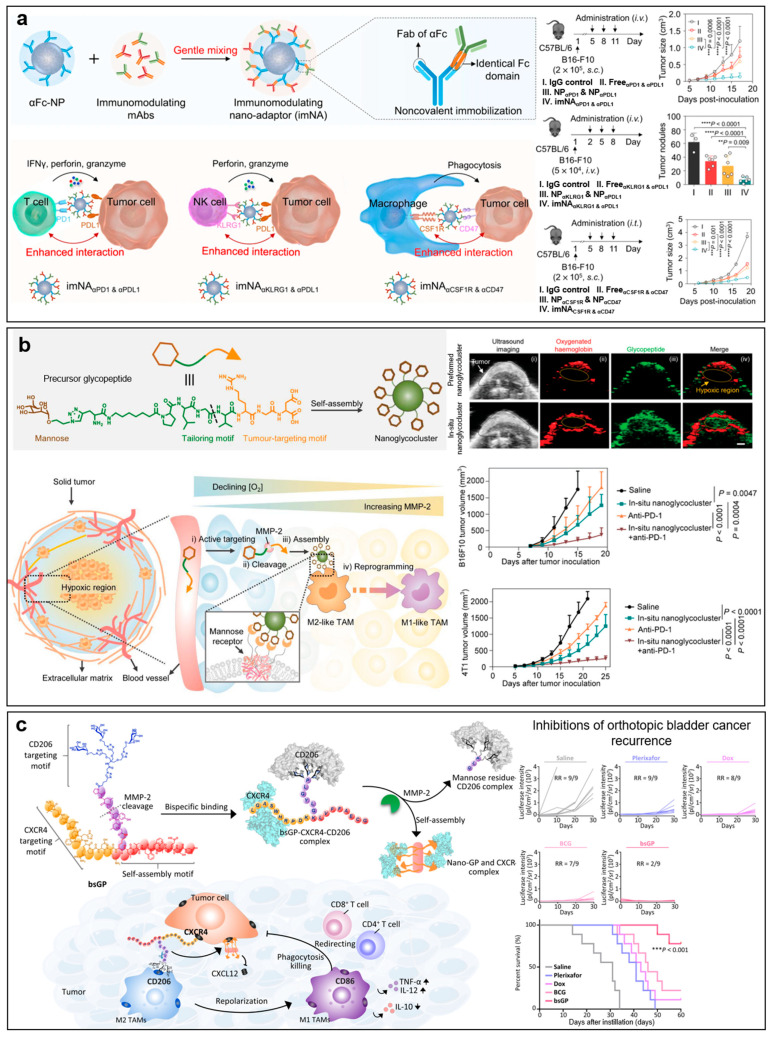
Immune-reprogramming nanomaterials for immunotherapy. (**a**) Immunomodulating nano-adaptors to promote antibody-based cancer immunotherapy. Figure adapted with permission from Ref. [398] under the Creative Commons CC BY license. Copyright 2021, Springer Nature. (**b**) In vivo self-assembled glycopeptide for reprogramming tumor-associated macrophages to boost cancer immunotherapy. Figures adapted with permission from Ref. [399]. Copyright 2023, Wiley-VCH GmbH. (**c**) Bispecific glycopeptide for spatiotemporal regulation of tumor microenvironment to inhibit bladder cancer recurrence. Figure adapted with permission from Ref. [400] under the Creative Commons CC BY license. Copyright 2023, American Association for the Advancement of Science.

To boost the therapeutic effects of immunotherapy, reprogramming immune cells demonstrated attractive potential. One of the most successful examples is the chimeric antigen receptor T cell (CAR T) therapy [396]. In this therapeutic, one’s own immune cells, mainly T cells and NK cells, are genetically engineered to express chimeric antigen receptors, allowing the immune cells to recognize and kill tumor cells specifically. Currently, six CAR T therapies have been approved for cancer therapy by the FDA since the first approval in 2017. However, CAR T therapy still encounters limitations such as patient dependence, clinical toxicities (e.g., cytokine release syndrome and neurotoxicity), and resistance. Besides cell-based therapy, immune-reprogramming nanomaterials have recently demonstrated genius in cancer immunotherapy. Unlike CAR T therapy, these nanomaterials can reprogram immune cells in the body without the need to extract immune cells. In addition, compared with direct delivery of drugs to tumors that encounter a high risk of clearance by the immune system, the immune-reprogramming nanocarriers are designed to target and re-activate immune cells. For instance, Nahmad et al. directly engineered B cells in vivo to secrete neutralizing anti-HIV antibodies in mice [397]. They prepared two adeno-associated viral vectors to encode *Staphylococcus aureus* Cas9 and broadly neutralizing antibody 3BNC117, respectively. Via the intravenous administrations of two vectors, B cells in mice were engineered to express high levels of broadly neutralizing antibodies at neutralizing titers of up to 6.8 µg ml^−1^. In addition to genetic engineering, self-assembled nanomaterials that directly modify the surface of immune cells have recently attracted much attention. For instance, Jiang et al. reported a type of immunomodulating nano-adaptors to promote antibody-based cancer immunotherapy (Figure 7a) [398]. These nano-adaptors comprised an anti-IgG (Fc specific)-modified polystyrene nanoparticle core and a shell consisting of two monoclonal antibodies. These antibodies were conjugated on the nanoparticle surface via Fc-specific noncovalent interactions. When conjugated with anti-PD-1 antibodies and anti-PD-L1 antibodies, the resulting nano-adapters effectively promoted T cell-tumor cell interactions and augmented the T cell-mediated immunotherapy in subcutaneous B16-F10 melanoma tumor-bearing mice. The average tumor volume in the nano-adapter group was 4.3-fold and 3.2-fold smaller than those receiving the mixture of two antibodies and the mixture of two single antibody-conjugated nanoparticles, respectively. Via conjugations of anti-killer-cell lectin-like receptor G1 antibodies and anti-PDL1 antibodies, the resulting nano-adapters enhanced the NK-cell mediated immunotherapy in B16-F10 pulmonary metastatic tumor-bearing mice. The metastatic nodules in the lungs of nano-adapter-treated mice (median, ~7) were much less than those of the mixture of two antibodies-treated mice (median, ~34), the mixture of two single antibody-conjugated nanoparticles-treated mice (median, ~27), and IgG control-treated mice (median, ~62), respectively. In addition, the nano-adapters with anti-factor 1-receptor antibodies and anti-CD47 antibodies could also improve macrophage-mediated immunotherapy by converting tumor-supportive M2 macrophages to tumoricidal M1 macrophages and physically connecting macrophages and tumor cells. Besides pre-assembled nanoparticles, Zhao et al. also reported an in vivo self-assembled glycopeptide for reprogramming tumor-associated macrophages (TAMs) to boost cancer immunotherapy (Figure 7b) [399]. The glycopeptide consisted of a tumor-targeting motif, an MMP-2 cleavable linker, and a mannose moiety for targeting mannose receptors on M2-like TAMs (peptide sequence: Mannose-alkyl-PLGVRGRGD). After systemic administration, the precursor glycopeptide entered tumor tissues via active targeting and was cleaved by MMP-2 enzymes. The resulting mannose segment further self-assembled into nanoparticles with improved binding affinity to the mannose receptors (411-fold decrease in the dissociation constant), leading to the switch of M2-like microphases to M1-like ones and enhancement in tumor penetration. Owing to the advantages of deep tumor penetration and enhanced hypoxic TAMs repolarization, this glycopeptide with anti-PD-1 antibody achieved a 90.2% tumor inhibitory rate in the TAMs-abundant 4T1-breast cancer model. Furthermore, to further improve the spatiotemporal specificity for immunotherapy, An et al. reported a bispecific glycopeptide that targeted CD206 on M2-like TAMs and CXCR4 receptors on tumor cells for inhibiting bladder cancer recurrence (Figure 7c) [400]. The peptide consisted of 4 modules, including (i) a CD206-targeting motif with (ii) an MMP-2 cleavable linker, (iii) a CXCR4-targeting motif, and (iv) a self-assembly motif (peptide sequence: LGASWHRPDKK(PLGYLG-(man)_3_)LVFFAECG). In the tumor microenvironment, the peptide repolarized M2-like TAMs to the M1 phenotype, promoting the recruitment of CD8^+^ T cells. Meanwhile, the peptide was cleaved by MMP-2 enzymes and formed CXCR4-binding nanofibers in situ for the long-term arrest of CXCR4 signaling, promoting T cell infiltration. Owing to the spatiotemporal regulation of tumor microenvironment, this bispecific glycopeptide reduced the recurrent rate of orthotopic bladder MB49-Luc tumor-bearing mice to 22% compared with 100% for saline and plerixafor groups and 89% for the DOX group.

## 5. Conclusions and Perspective

In this review, we briefly presented recent progress on multistage self-assembled nanomaterials for cancer immunotherapy. The traditional “one size fits all” approach can hardly confront the sophisticated physiological and tumoral environments. Therefore, nanomaterials that can overcome multiple biological barriers are highly desirable. Among them, in situ self-assembled nanomaterials have attracted much attention, allowing precise and on-demand self-assembly in disease sites triggered by internal and external stimuli. These nanomaterials have displayed advantages such as prolonged blood circulation, enhanced tumor accumulation and penetration, increased tumor cell internalization, controlled drug release in specific sites, and improved immune responses. These features enable them to achieve improved cancer therapeutics with reduced side effects. Despite significant advancements in multistage self-assembled nano-delivery systems, key challenges associated with nanomedicines persist. These include inadequate specific targeting, limited delivery efficacy, and potential side effects. Several critical issues should be considered for the clinical success of these smart nanomaterials.

First, the multistage nanocarriers involve multiple and programmed delivery procedures in the body. Reliable and non-invasive monitoring techniques or multimode tracking probes for evaluating the process and efficacy for each stage are highly desired. Second, for many multistage nanocarriers, their architectures and constructions are highly complicated. Concerns regarding reproducibility and high quality control in scale-up manufacturing must be addressed. Third, the safety and side effects of the multistage nanocarriers should be highly considered, particularly the immune-related side effects and long-term toxicity. Fourth, human immune systems are quite different from experimental animal models. Developing reliable humanized animal models and organs-on-chips with immune systems is urgent. Fifth, a fundamental and deep understanding of the thermodynamics and kinetics of in vivo self-assembly is critical to spatiotemporally control of the self-assembly process, assembled structures, distribution, retention, and excretion in the body. Last but not least, discovering novel biomaterials using artificial intelligence is the new research paradigm in biomedical fields. High-throughput material library and screening are urgent to build up the structure-activity relationships in biomaterialomics to guide the rational material design of nanomaterials for immunotherapy driven by machine learning and data science. Bridging together the cutting edges of nanotechnology, biotechnology, and data science, multistage self-assembled nanomaterials are expected to prompt the advent of precise and efficient cancer therapy in the near future.

## Figures and Tables

**Table 1:** Currently marketed anticancer nanomedicines in the clinic.

Generic Name	Formulations	Active Pharmaceutical Ingredients	Cancer Type	Approved Year
Doxil	Pegylated liposome	Doxorubicin	HIV-related Kaposi sarcoma, ovarian cancer, and multiple myeloma	1995
DaunoXome	Liposome	Daunorubicin	HIV-related Kaposi sarcoma	1996
Myocet	Liposome	Doxorubicin	Metastatic breast cancer	2000 (Europe)
Lipusu	Liposome	Paclitaxel	Breast cancer and non-small-cell lung cancer	2003 (China)
Mepact	Liposome	Muramyl tripeptide phosphatidylethanolamine	Nonmetastatic, resectable osteosarcoma	2009 (Europe)
Marqibo	Liposome	Vincristine sulfate	Acute lymphoblastic leukemia	2012
Onivyde	Pegylated liposome	Irinotecan	Post-gemcitabine metastatic pancreatic cancer	2015
Liporaxel	Lipid nanoparticle (oral)	Paclitaxel	Gastric cancer	2016 (Korea)
Vyxeos	Liposome	Daunorubicin and cytarabine	Secondary acute myeloid leukemia	2017
Abraxane	Albumin-bond nanoparticles	Paclitaxel	Breast, lung, and pancreatic cancer	2005
SMANCS	Polymer conjugate	Neocarzinostatin	Liver and renal cancer	1993 (Japan)
Genexol-PM	Polymeric micelle	Paclitaxel	Breast cancer and non-small cell lung cancer	2007 (Korea)
PICN	Polymeric nanoparticle	Paclitaxel	Breast cancer	2014 (India)
Apealea/Paclical	Small molecular (XR-17) micelle	Paclitaxel	Ovarian cancer	2015 (Russia)/2018 (Europe)
NanoTherm	Inorganic nanoparticle	Iron oxide nanoparticle-induced hyperthermia	Glioblastoma	2010 (Europe)
NBTXR3	Inorganic nanoparticle	Hafnium oxide nanoparticles	Locally advanced squamous cell carcinoma	Fast track designation in 2020

HIV: human immunodeficiency virus.

**Table 2:** Recent examples of stimuli-responsive nanosystems for cancer immunotherapy.

Stimulus	Delivery Formulation	Responsive Module	Therapeutic Agents	Tumor Model	Ref.
pH	Albumin nanoparticles containing Cu_2_(OH)_2_CO_3_ nanocrystals	Cu_2_(OH)_2_CO_3_ nanocrystals	Cu^2+^, disulfiram, and anti-PD-L1 antibody	Orthotopic 4T1 tumor	[287]
	Polymeric nanoparticles composed of PCL-b-PEG	Hydrazone bond	HCP antigen and CpG ODN	Subcutaneous EMT6 and 4T1 tumors	[288]
	CaCO_3_ nanoparticles	CaCO_3_	CpG ODN, INCB24360 (IDO inhibitor), and Ca^2+^	Subcutaneous 4T1-Luc tumor	[289]
	Polymeric nanoparticles composed of PEG-b-PDPA	4-Acetoxybenzyl ester bond	DMXAA (STING agonist), neoantigens, and anti-PD-L1 antibody	Subcutaneous 4T1 tumor	[290]
	Polymeric nanoparticles composed of RGD-PEG-b-PGA-g-(TETA-DTC-PHis)	Benzoic-imine bond and histidine moiety	Resiquimod (R848, TLR 7/8 agonist)	Metastatic 4T1 tumor	[291]
	Cocktail polymeric nanoparticles, including DOX-loaded PLG-g-PEG nanoparticles and nanoparticles composed of RNA-loaded OHC-PEG-CHO, PLG, and PEI	Glutamic acid residue and Schiff bases formed between amino groups and aldehyde groups	DOX and small hairpin RNA of PD-L and hyaluronidase	Subcutaneous B16F10, 4T1, and CT26 tumors	[292]
	Antibody-pH low insertion peptide conjugate (peptide sequence: Ac-ACEQNPIYWARYADWLFTTPLLLLDLALLVDADEGT)	pH low insertion peptide	Anti-CD20 antibody (activator for NK cell-mediated cytotoxicity)	Subcutaneous B16 F10 and 4T1 tumors, and metastasis 4T1 tumor	[293]
ROS	Albumin–antibody complex	2,2′-[Propane-2,2-diylbis(thio)]diacetic acid	Anti-CD47 antibody and anti-PD-1 antibody	Subcutaneous B16F10-Luc tumor	[294]
	Peptide-based gel depot	L-methionine residues	Anti-PD-1 antibody and D-1MT (IDO inhibitor)	Subcutaneous B16F10-tumor	[295]
	Polymeric nanoparticles composed of aspirin-dextran conjugates	4-Formylbenzeneboronic acid pinacol ester	Aspirin (COX-2 inhibitor) and anti-PD-1 antibody	Subcutaneous CT26 tumor	[296]
	Polymeric nanoparticles composed of chitosan modified with PEG-T7 peptide (peptide sequence: HAIYPRH)	4-Nitrophenyl-4-(4,4,5,5-tetramethyl-1,3,2-dioxaborolan-2-yl) benzyl carbonate	DOX and siRNA-PD-L1	Subcutaneous 4T1 tumor	[297]
	Nanoparticles composed of pemetrexed and β-seleno ester	β-Seleno ester	Pemetrexed and β-seleno ester	Subcutaneous A549 tumor	[298]
	Diselenide-bridged organosilica nanoparticles	Diselenide-bond	Annexin A5	Orthotopic 4T1-Luc tumor, and subcutaneous B16F10-Luc and CT-26 tumors	[219]
Enzyme	Polymeric nanoparticles composed of PEG-peptide-IDO inhibiter conjugates	MMP-2 responsive peptide (sequence: PVGLIG)	Epacadostat (IDO inhibitor) and ICG (photosensitizer)	Subcutaneous B16-F10 tumor	[299]
	Triglycerol monostearate nanoparticles containing Pd nanoparticles and DOX	MMP-2 responsive triglycerol monostearate	DOX and Pd nanoparticles (photothermal agents)	Subcutaneous CT26 tumor	[300]
	Polymeric nanoparticles composed of PLL-1-mt and HA-Ce6	Hyaluronidase-responsive hyaluronic acid	Anti-PD-L1 antibody, 1-methyl tryptophan (IDO inhibitor), and Ce6 (photosensitizer)	Subcutaneous and metastatic B16-F10 tumors	[301]
	Nanoparticles composed of TPT-conjugated PLLA as core and HA-DOX as shell	Hyaluronidase-responsive hyaluronic acid	Anti-PD-L1 antibody and DOX	Subcutaneous 4T1-Luc tumor	[302]
	Peptide-based nanoparticles	MMP-2 responsive peptide (sequence: PLGLAG)	Anti-PD-L1 peptide and IR780 (photosensitizer)	Subcutaneous B16-F10 tumor	[303]
	Nanoparticles composed of PEG-GALGLPG-PPa, DPPC, and lipid-mimetic NLG919 prodrug	MMP-2 responsive peptide (sequence: PLGLAG)	Pyropheophorbide-a (photosensitizer) and NLG919 (IDO-1 inhibitor)	Subcutaneous CT26 and 4T1 tumors	[304]
Hypoxia	Mesoporous silica nanoparticles	Azobenzene linker	Ce6 and CpG ODN	Subcutaneous B16.F1 tumor	[305]
	Nanovesicles composed of manganese ferrite nanoparticles grafted with hypoxia-responsive PEG-b-PNIHM	2-Nitroimidazoles	Anti-PD-L1 antibody, DOX, and manganese ferrite nanoparticles (converting H_2_O_2_ to O_2_)	Subcutaneous 4T1 tumor	[306]
	IFN-poly(N-oxide) conjugates	Poly(N-oxide) moiety	IFN	Subcutaneous C8161 tumor	[307]
	Nanoparticles composed of AIEgen, hypoxia-responsive paclitaxel prodrug, Pluronic F127, and M1 macrophage cell membrane as shell	4-Nitrobenzyl carbonate moiety	AIEgen (photodynamic therapy) and paclitaxel	Subcutaneous 4T1 tumor	[308]
	Polymeric nanoparticles composed of PEG-b-P(Asp-g-NIDH), OTS964, and Ce6	2-Nitroimidazole	OTS964 (TOPK inhibitor) and Ce6	Subcutaneous KYSE 150 tumor	[226]
NIR	Nanoparticles composed of PD-L1 aptamer-functionalized MOF	Porphyrinic Zr_6_ MOF	Zr_6_ MOF, PD-L1 aptamer, and oxaliplatin	Subcutaneous Mc38 tumor	[309]
	Biosynthesized gold nanoparticles (Ausome)	Ausome	Ausome (generating hyperthermia under laser irradiation, improving tissue blood perfusion, and contributing to enhanced infiltration of immunostimulatory modules)	Orthotopic 4T1 tumor	[310]
	Hydrogels composed of Pd SAzyme, camptothecin, and agarose	Pd SAzyme	Camptothecin and Pd SAzyme (converting light to heat and H_2_O_2_ to •OH)	Subcutaneous CT26 tumor	[311]
	Photothermal conjugated polymeric nanoparticles	Diketopyrrolopyrrole units in conjugated polymers	Conjugated polymers and heat-activated IFN-γ plasmid	4T1 cancer cells	[312]
	Upconversion nanoparticles	ICG	Anti-CTLA-4 antibody, ICG (light absorber), rose Bengal (photosensitizer), and DSPE-PEG-maleimide (antigen-capturing agent)	Orthotopic 4T1 tumor	[313]
Ultrasound	TiO_2_@CaP core–shell nanoparticles	Acid-responsive CaP shell and sonosensitizer TiO_2_ nanoparticle	Anti-PD-1 antibody and TiO_2_ nanoparticle	Subcutaneous 4T1 tumor	[314]
	Semiconducting polymeric nanoparticles	Semiconducting polymer	Semiconducting polymer (generateing ^1^O_2_ under ultrasound irradiation), NLG919, and anti-PD-L1 antibody	Subcutaneous Panc02 tumor and orthotopic rabbit pancreatic tumor model using VX2 tumor cells	[315]
	Crosslinked nanoparticles composed of hematoporphyrin, adenosine deaminase, anti-PD-L1 antibody, and bovine serum albumin	Sonosensitizer hematoporphyrin, acid-cleavable imine bond, and ROS-cleavable thioketal bonds	Hematoporphyrin (generating ^1^O_2_ under ultrasound irradiation), anti-PD-L1 antibody, and adenosine deaminase	Subcutaneous 4T1 and CT26 tumors	[316]
	Self-healing hydrogel	Hydrogel polymerized from OEGMA as monomer and inorganic clay as cross-linker	OVA, imiquimod (R837, immune adjuvant), and anti-PD-L1 antibody	Subcutaneous B16-OVA and orthotopic 4T1-Luc tumors	[317]
	Engineered bacteria	Focused ultrasound to generate heat in tumor tissue	Engineered bacteria with a temperature-actuated genetic state switch to produce anti-CTLA-4 and anti-PD-L1 antibodies	Subcutaneous A20 tumor	[318]
	Engineered bacteria	Focused ultrasound to generate heat in tumor tissue	Engineered bacteria with a temperature-actuated genetic state switch to produce IFN-γ	Subcutaneous 4T1 tumor	[319]
Radiation	Cancer cell membrane-coated mesoporous organosilica nanoparticles	Diselenide bond	DOX and anti-PD-L1 antibody	Orthotopic 4T1 tumor	[320]
	Nanoparticles prepared from pemetrexed and cytosine-containing diselenide	Diselenide bond	Pemetrexed and diselenide species	Subcutaneous MDA-MB-231 tumor	[321]
	Polymeric nanoparticles prepared from selenium-containing polymer	Diselenide bond	DOX and diselenide species	Subcutaneous MDA-MB-231 tumor	[322]
	Se/Te nanochaperone	Se/Te nano-heterojunctions	Se/Te nanochaperone	Subcutaneous 4T1 tumor	[323]

HCP: heat shock protein 70 (HSP70)-chaperoned polypeptides; PCL-b-PEG: poly(ε-caprolactone)-b-poly(ethylene glycol); CpG ODN: CpG oligodeoxynucleotide; IDO: indoleamine-2,3-dioxygenase; DMXAA: 5,6-dimethylxanthenone-4-acetic acid; PEG-b-PDPA: PEG-b-poly(2-(diisopropanol amino) ethyl methacrylate); RGD-PEG-b-PGA-g-(TETA-DTC-PHis): RGD-PEG-b-PGA-g-(triethylenetetramine-bis(dithiocarbamate)-poly-L-histidine; TLR: Toll-like receptor; PLG-g-PEG: poly(L-glutamic acid)-g-PEG; OHC-PEG-CHO: aldehyde-modified polyethylene glycol; PEI: polyethylenimine; D-1MT: dextro-1-methyl tryptophan; MMP-2: matrix metalloproteinase-2; COX-2: cyclooxygenase-2; ICG: indocyanine green; PLL-1-mt: dextro-1-methyl tryptophan-conjugated poly(L-lysine); HA-Ce6: Chlorin e6 (Ce6)-conjugated hyaluronic acid; TPT: triphenylphosphine; PLLA: poly(L-lactic acid); HA-DOX: DOX decorated hyaluronic acid; PEG-GALGLPG-PPa: PEG-GALGLPG-pyropheophorbide-a conjugates; DPPC: 1,2-dipalmitoyl-sn-glycero-3-phosphocholine; PEG-b-PNIHM: PEG-*b*-poly(6-(2-nitroimidazol-1-yl)hexyl methacrylate; IFN: interferon alpha; AIEgen: aggregation-induced emission luminogen; TOPK: T-lymphokine-activated killer cell-originated protein kinase; PEG-b-P(Asp-g-NIDH): PEG-*b*-poly[aspartic acid-graft-6-(2-nitroimidazole)hexylamine]; MOF: metal-organic framework; SAzyme: single-atom nanozyme; DSPE-PEG-maleimide: 1, 2-distearoyl-sn-glycero-3-phosphoethanolamine-PEG-maleimide; OVA: ovalbumin; OEGMA: oligo (ethylene glycol) methacrylate. 4T1 and EMT6: murine breast tumor cell lines; B16F10, B16.F1, and B16-OVA: murine melanoma cell lines; CT26: murine colorectal tumor cell line; C8161: human melanoma cell line; KYSE 150: human esophageal squamous cell carcinoma cell line; Mc38: murine colon adenocarcinoma cell line; Panc02: murine pancreatic tumor cell line; VX2: rabbit liver tumor cell line; A20: murine lymphoma tumor cell line; MDA-MB-231: human breast tumor cell line; A549: human non-small cell lung cancer cell line.

**Table 3:** Recent examples of multistage self-assembled nanocarriers for cancer immunotherapy.

Strategy	Nanomaterial Formulation	Therapeutic Agent	Delivering Stages	Tumor Model	Ref.
Programmable delivery	Nanoparticles composed of Fe_3_O_4_-Au as core with mesoporous silica shell and surface modification of enzyme cleavable therapeutic peptides	Methylene blue (photosensitizer) and PD-L1 blocking peptide P^D^PPA-1	Initial: nanoparticles (~220 nm); in tumor tissues: the peptide corona is cleaved by MMP-2 enzyme and GSH, resulting in the release of PD-L1 blocking peptide, shrinkage of nanoparticle size (to less than 100 nm), and surface charge conversion to improve cell uptake; in the cytoplasm: the methylene blue is released to produce ROS under 660 nm laser irradiation.	Subcutaneous EMT6 tumor	[353]
	Polymeric nanoparticles composed of Pt(IV) prodrug-conjugated PEG-b-PHEP, TK-PPE, Ce6, and BLZ-945	Ce6 (producing ROS under laser irradiation to cleave thioketal bond), BLZ-945 (CSF1R inhibitor), and Pt(IV) drug	Initial: nanoparticles (~280 nm); in tumor tissues under 660 nm laser: the nanoparticle size is shrunk to ~70 nm due to the cleavage of thioketal bond for deep penetration to kill tumor cells. Meanwhile, BLZ-945 is released for depleting TAMs.	Subcutaneous 4T1 and CT26 tumors	[354]
	Semiconducting polymeric nanoparticles decorated enzyme-cleavable PROTAC peptides	Semiconducting polymer (generating ^1^O_2_) under NIR irradiation) and IDO-targeting PROTAC peptide	Initial: nanoparticles (~30 nm); in tumor tissues and cells under 808 nm laser: semiconducting polymer generates ^1^O_2_ to eradicate tumor cells for inducing ICD; In tumor cells: IDO-targeting PROTAC peptides are cleaved by cathepsin B enzymes to degrade IDO and promote immunotherapy.	Subcutaneous 4T1 tumor	[355]
	Nanoparticles composed of DiPt-TK-PEG and NLG919-disulfide linker-PPa	PPa (generating ROS under NIR light), oxaliplatin, NLG919	Initial: nanoparticles (~112 nm); in tumor tissues under the first wave of laser (671 nm) irradiation: PPa generates ROS to cleave thioketal linker for PEG corona detachment, promoting tumor retention and deep penetration; in tumor cells: nanoparticles are decomposed triggered by GSH to release PPa, NLG919, and oxaliplatin. Under the second wave of laser irradiation, PPa produce produces ROS in combination with oxaliplatin to induce ICD. Meanwhile, NLG919 reverses the immunosuppressive tumor microenvironment by suppressing IDO-1-mediated tryptophan degradation and cytotoxic T lymphocyte exhaustion.	Subcutaneous and metastatic 4T1 tumors.	[356]
In vivo self-assembled nanomaterials	Self-assembled bispecific peptide (sequence: AKMGEGGWGANDY-GNNQQNY-RGD)	Integrin-targeting peptide (RDG) and CD3-targeting peptide (AKMGEGGWGANDY)	Initial: isolated peptides; in tumor tissues: receptor-induced clustering of self-assembled peptides occurs in situ to active T cells.	MCF-7 cancer cells	[357]
	Polymer-peptide conjugates	Antigenic peptide and anti-PD-L1 antibody	Initial: nanospheres (~100 nm); in the acidic endosomal environment: nanoparticles transform into nanosheets (several micrometers in length or width), enhancing delivery efficacy of antigenic peptides.	Subcutaneous B16F10-OVA and HPV-E6/E7 tumors	[206]
	In situ-formed hydrogel composed of PVA crosslinked by ROS-labile linker TSPBA	Gemcitabine and anti-PD-L1 antibody	Initial: injectable solution; in tumor tissues: peptide form hydrogel formation in situ and sustained release encapsulated gemcitabine to enhance an immunogenic tumor phenotype and anti-PD-L1 antibody to promote therapeutic immune response.	Subcutaneous B16F10 and 4T1 tumors	[358]
	Self-assembled modular peptide (sequence: SSGGPLGVRGKLVFFCAWSATWSNYWRH)	CD47 blocking peptide (CAWSATWSNYWRH) and anti-PD-L1 antibody	Initial: isolated peptides; in tumor tissues: peptides target CD47 on tumor cell membranes and are cleaved by MMP-2 enzymes to form nanofibers in situ to block CD47, promoting the activation of TAMs.	Subcutaneous LLC tumor	[359]
	Phthalocyanine derivative (PcN4)	PcN4, AQ4N (hypoxia-activated cytotoxin prodrug), and anti-PD-L1 antibody	Initial: isolated PcN4; in bloodstream: PcN4 interacts with endogenous albumin dimers and forms supramolecular complexes; in primary tumor tissues: concomitant delivery of AQ4N ameliorates the limitation of hypoxia in photodynamic therapy of PcN4 complexes, promoting anticancer efficacy and activation of CD^8+^ T cells; in distance tumor: additional combination therapy using the anti-PD-L1 antibody.	Orthotopic 4T1 tumor	[360]
Immune-reprogramming nanomaterials	DNA nanostructures with spatial precision in immune stimulating ligand	CD3ɛ antibodies, CD28 antibodies, and T cells	In vitro: T cells are activated and expanded by DNA origami with CD3ɛ antibodies that stimulate TCR ligands and CD28 antibodies that simulate co-stimulatory ligands, with inter-ligand spacing from ∼95 to ∼16 nm. A space of ∼38 nm between TCR ligands and co-stimulatory ligands is appropriate for efficient T cell activation; in vivo: T cell adaptive transfer for immunotherapy.	Subcutaneous B16-OVA tumor	[361]
	DNA-engineered red blood cells-based artificial antigen-presenting cells	Engineered red blood cells modified with pMHC and anti-CD28 antibody, as well as splenocytes from OT-1 mice	In vitro: surface engineering of red blood cells by modification with lipid-DNA, clustered distributed pMHC, and anti-CD28 antibody sequentially; in vivo: reinfusion of the resultant artificial antigen-presenting cells for tumor immunotherapy together with OT-1 splenocytes.	Subcutaneous B16-OVA tumor	[362]
	DNA-engineered lymphocyte-based artificial antigen-presenting cells	Engineered lymphocytes modified with pMHC and anti-CD28 antibody, as well as anti-PD-1 antibody	In vitro: surface engineering of lymphocytes collected from peripheral blood by modification with lipid-DNA, clustered distributed pMHC, and anti-CD28 antibody sequentially; in vivo: reinfusion of the resultant artificial antigen-presenting cells for tumor immunotherapy together with anti-PD-1 antibody.	Subcutaneous B16-OVA and Mc38 tumors	[363]

GSH: glutathione; PEG-*b*-PHEP: PEG-*b*-poly (2-hexoxy-2-oxo-1,3,2-dioxaphospholane); TK-PPE: poly(thioketal phosphoester); CSF1R: colony-stimulating factor 1 receptor; TAMs: tumor-associated macrophages; PROTAC: proteolysis targeting chimera; ICD: immunogenic cell death; DiPt-TK-PEG: PEG-thioketal linker-oxaliplatin; PPa: pheophorbide A; MCF-7: human breast cancer cell line; HPV: human papilloma virus; PVA: poly(vinyl alcohol); TSPBA: N^1^-(4-boronobenzyl)-N^3^-(4-boronophenyl)-N^1^,N^1^,N^3^,N^3^-tetramethylpropane-1,3-diaminium; TCR: T cell receptor; pMHC: peptide–major histocompatibility complex. LLC: murine Lewis lung carcinoma cell line.

## Data Availability

Data are contained within the article.

## References

[1] Demaria O., Cornen S., Daeron M., Morel Y., Medzhitov R., Vivier E. (2019). Harnessing innate immunity in cancer therapy. Nature.

[2] Sanoff H.K. (2022). Improving Treatment Approaches for Rectal Cancer. N. Engl. J. Med..

[3] Obradovic A. (2023). Precision immunotherapy. Science.

[4] He X., Xu C. (2020). Immune checkpoint signaling and cancer immunotherapy. Cell Res..

[5] Peng S., Xiao F., Chen M., Gao H. (2022). Tumor-Microenvironment-Responsive Nanomedicine for Enhanced Cancer Immunotherapy. Adv. Sci..

[6] Binnewies M., Roberts E.W., Kersten K., Chan V., Fearon D.F., Merad M., Coussens L.M., Gabrilovich D.I., Ostrand-Rosenberg S., Hedrick C.C. (2018). Understanding the tumor immune microenvironment (TIME) for effective therapy. Nat. Med..

[7] Zhang Y., Zhang Z. (2020). The history and advances in cancer immunotherapy: Understanding the characteristics of tumor-infiltrating immune cells and their therapeutic implications. Cell Mol. Immunol..

[8] Murciano-Goroff Y.R., Warner A.B., Wolchok J.D. (2020). The future of cancer immunotherapy: Microenvironment-targeting combinations. Cell Res..

[9] Waldman A.D., Fritz J.M., Lenardo M.J. (2020). A guide to cancer immunotherapy: From T cell basic science to clinical practice. Nat. Rev. Immunol..

[10] Lin M.J., Svensson-Arvelund J., Lubitz G.S., Marabelle A., Melero I., Brown B.D., Brody J.D. (2022). Cancer vaccines: The next immunotherapy frontier. Nat. Cancer.

[11] Ribas A., Wolchok J.D. (2018). Cancer immunotherapy using checkpoint blockade. Science.

[12] Hodi F.S., O’Day S.J., McDermott D.F., Weber R.W., Sosman J.A., Haanen J.B., Gonzalez R., Robert C., Schadendorf D., Hassel J.C. (2010). Improved survival with ipilimumab in patients with metastatic melanoma. N. Engl. J. Med..

[13] Doroshow D.B., Bhalla S., Beasley M.B., Sholl L.M., Kerr K.M., Gnjatic S., Wistuba I.I., Rimm D.L., Tsao M.S., Hirsch F.R. (2021). PD-L1 as a biomarker of response to immune-checkpoint inhibitors. Nat. Rev. Clin. Oncol..

[14] Kennedy L.B., Salama A.K.S. (2020). A review of cancer immunotherapy toxicity. CA Cancer J. Clin..

[15] Cabral H., Miyata K., Osada K., Kataoka K. (2018). Block Copolymer Micelles in Nanomedicine Applications. Chem. Rev..

[16] Chen H.B., Gu Z.J., An H.W., Chen C.Y., Chen J., Cui R., Chen S.Q., Chen W.H., Chen X.S., Chen X.Y. (2018). Precise nanomedicine for intelligent therapy of cancer. Sci. China Chem..

[17] Wei G.Q., Wang Y., Huang X.H., Hou H.B., Zhou S.B. (2018). Peptide-Based Nanocarriers for Cancer Therapy. Small Methods.

[18] AlSawaftah N.M., Awad N.S., Pitt W.G., Husseini G.A. (2022). pH-Responsive Nanocarriers in Cancer Therapy. Polymers.

[19] Shi J., Kantoff P.W., Wooster R., Farokhzad O.C. (2017). Cancer nanomedicine: Progress, challenges and opportunities. Nat. Rev. Cancer.

[20] Wang J., Li Y., Nie G. (2021). Multifunctional biomolecule nanostructures for cancer therapy. Nat. Rev. Mater..

[21] Chaudhuri O., Cooper-White J., Janmey P.A., Mooney D.J., Shenoy V.B. (2020). Effects of extracellular matrix viscoelasticity on cellular behaviour. Nature.

[22] He M., Yang T., Wang Y., Wang M., Chen X., Ding D., Zheng Y., Chen H. (2021). Immune Checkpoint Inhibitor-Based Strategies for Synergistic Cancer Therapy. Adv. Healthc. Mater..

[23] Gao J., Wang W.Q., Pei Q., Lord M.S., Yu H.J. (2020). Engineering nanomedicines through boosting immunogenic cell death for improved cancer immunotherapy. Acta Pharmacol. Sin..

[24] Yi Y., Lin G., Chen S., Liu J., Zhang H., Mi P. (2018). Polyester micelles for drug delivery and cancer theranostics: Current achievements, progresses and future perspectives. Mater. Sci. Eng. C.

[25] Ekladious I., Colson Y.L., Grinstaff M.W. (2019). Polymer-drug conjugate therapeutics: Advances, insights and prospects. Nat. Rev. Drug Discov..

[26] Yi Y., An H.W., Wang H. (2023). Intelligent Biomaterialomics: Molecular Design, Manufacturing, and Biomedical Applications. Adv. Mater..

[27] Begines B., Ortiz T., Perez-Aranda M., Martinez G., Merinero M., Arguelles-Arias F., Alcudia A. (2020). Polymeric Nanoparticles for Drug Delivery: Recent Developments and Future Prospects. Nanomaterials.

[28] Sung Y.K., Kim S.W. (2020). Recent advances in polymeric drug delivery systems. Biomater. Res..

[29] Pattni B.S., Chupin V.V., Torchilin V.P. (2015). New developments in liposomal drug delivery. Chem. Rev..

[30] Liu P., Chen G., Zhang J. (2022). A Review of Liposomes as a Drug Delivery System: Current Status of Approved Products, Regulatory Environments, and Future Perspectives. Molecules.

[31] Wang S., Chen Y., Guo J., Huang Q. (2023). Liposomes for Tumor Targeted Therapy: A Review. Int. J. Mol. Sci..

[32] Samaridou E., Heyes J., Lutwyche P. (2020). Lipid nanoparticles for nucleic acid delivery: Current perspectives. Adv. Drug Deliv. Rev..

[33] Hou X., Zaks T., Langer R., Dong Y. (2021). Lipid nanoparticles for mRNA delivery. Nat. Rev. Mater..

[34] Tenchov R., Bird R., Curtze A.E., Zhou Q. (2021). Lipid Nanoparticles horizontal line From Liposomes to mRNA Vaccine Delivery, a Landscape of Research Diversity and Advancement. ACS Nano.

[35] Luo Z., Gao Y., Duan Z., Yi Y., Wang H. (2021). Mitochondria-Targeted Self-Assembly of Peptide-Based Nanomaterials. Front. Bioeng. Biotechnol..

[36] Li L.L., Qiao Z.Y., Wang L., Wang H. (2019). Programmable Construction of Peptide-Based Materials in Living Subjects: From Modular Design and Morphological Control to Theranostics. Adv. Mater..

[37] Qi G.B., Gao Y.J., Wang L., Wang H. (2018). Self-Assembled Peptide-Based Nanomaterials for Biomedical Imaging and Therapy. Adv. Mater..

[38] Chang R., Zhao L., Xing R., Li J., Yan X. (2023). Functional chromopeptide nanoarchitectonics: Molecular design, self-assembly and biological applications. Chem. Soc. Rev..

[39] Li S., Zhang W., Xue H., Xing R., Yan X. (2020). Tumor microenvironment-oriented adaptive nanodrugs based on peptide self-assembly. Chem. Sci..

[40] Zhang S., Lou X.Y., Liu L., Yang Y.W. (2023). The Creation of DNA Origami-Based Supramolecular Nanostructures for Cancer Therapy. Adv. Healthc. Mater..

[41] Ma W., Zhan Y., Zhang Y., Mao C., Xie X., Lin Y. (2021). The biological applications of DNA nanomaterials: Current challenges and future directions. Signal Transduct. Target. Ther..

[42] Dey S., Fan C., Gothelf K.V., Li J., Lin C., Liu L., Liu N., Nijenhuis M.A.D., Saccà B., Simmel F.C. (2021). DNA origami. Nat. Rev. Methods Primers.

[43] Kakkar A., Traverso G., Farokhzad O.C., Weissleder R., Langer R. (2017). Evolution of macromolecular complexity in drug delivery systems. Nat. Rev. Chem..

[44] Palmerston Mendes L., Pan J., Torchilin V.P. (2017). Dendrimers as Nanocarriers for Nucleic Acid and Drug Delivery in Cancer Therapy. Molecules.

[45] Moreira D.A., Santos S.D., Leiro V., Pego A.P. (2023). Dendrimers and Derivatives as Multifunctional Nanotherapeutics for Alzheimer’s Disease. Pharmaceutics.

[46] Rastogi V., Yadav P., Porwal M., Sur S., Verma A. (2022). Dendrimer as nanocarrier for drug delivery and drug targeting therapeutics: A fundamental to advanced systematic review. Int. J Polym. Mater. Polym. Bioma..

[47] Wang J., Li B., Qiu L., Qiao X., Yang H. (2022). Dendrimer-based drug delivery systems: History, challenges, and latest developments. J. Biol. Eng..

[48] Kamegawa R., Naito M., Miyata K. (2018). Functionalization of silica nanoparticles for nucleic acid delivery. Nano Res..

[49] Li W., Liu Z., Fontana F., Ding Y., Liu D., Hirvonen J.T., Santos H.A. (2018). Tailoring Porous Silicon for Biomedical Applications: From Drug Delivery to Cancer Immunotherapy. Adv. Mater..

[50] Simon-Yarza T., Mielcarek A., Couvreur P., Serre C. (2018). Nanoparticles of Metal-Organic Frameworks: On the Road to In Vivo Efficacy in Biomedicine. Adv. Mater..

[51] Mirkin C.A., Petrosko S.H. (2018). Spherical nucleic acids: Adding a new dimension to nucleic acids and clinical chemistry. Clin. Chem..

[52] Zheng X., Wu Y., Zuo H., Chen W., Wang K. (2023). Metal Nanoparticles as Novel Agents for Lung Cancer Diagnosis and Therapy. Small.

[53] Theivendran S., Lazarev S., Yu C. (2023). Mesoporous silica/organosilica nanoparticles for cancer immunotherapy. Exploration.

[54] Zhou H., Ge J., Miao Q., Zhu R., Wen L., Zeng J., Gao M. (2020). Biodegradable Inorganic Nanoparticles for Cancer Theranostics: Insights into the Degradation Behavior. Bioconjug. Chem..

[55] Saeb M.R., Rabiee N., Mozafari M., Verpoort F., Voskressensky L.G., Luque R. (2021). Metal-Organic Frameworks (MOFs) for Cancer Therapy. Materials.

[56] Janjua T.I., Cao Y., Yu C., Popat A. (2021). Clinical translation of silica nanoparticles. Nat. Rev. Mater..

[57] Fang R.H., Kroll A.V., Gao W., Zhang L. (2018). Cell Membrane Coating Nanotechnology. Adv. Mater..

[58] Chen Z., Wang Z., Gu Z. (2019). Bioinspired and biomimetic nanomedicines. Acc. Chem. Res..

[59] Lin Q., Peng Y., Wen Y., Li X., Du D., Dai W., Tian W., Meng Y. (2023). Recent progress in cancer cell membrane-based nanoparticles for biomedical applications. Beilstein J. Nanotechnol..

[60] Zhu L., Yu X., Cao T., Deng H., Tang X., Lin Q., Zhou Q. (2023). Immune cell membrane-based biomimetic nanomedicine for treating cancer metastasis. Acta Pharm. Sin. B.

[61] Fang R.H., Gao W., Zhang L. (2023). Targeting drugs to tumours using cell membrane-coated nanoparticles. Nat. Rev. Clin. Oncol..

[62] Zhang M., Hu S., Liu L., Dang P., Liu Y., Sun Z., Qiao B., Wang C. (2023). Engineered exosomes from different sources for cancer-targeted therapy. Signal Transduct. Target. Ther..

[63] Wang J., Zhu M., Nie G. (2021). Biomembrane-based nanostructures for cancer targeting and therapy: From synthetic liposomes to natural biomembranes and membrane-vesicles. Adv. Drug Deliv. Rev..

[64] Luo G.F., Chen W.H., Zeng X., Zhang X.Z. (2021). Cell primitive-based biomimetic functional materials for enhanced cancer therapy. Chem. Soc. Rev..

[65] Zeng Z.L., Pu K.Y. (2020). Improving Cancer Immunotherapy by Cell Membrane-Camouflaged Nanoparticles. Adv. Funct. Mater..

[66] Ragelle H., Danhier F., Preat V., Langer R., Anderson D.G. (2017). Nanoparticle-based drug delivery systems: A commercial and regulatory outlook as the field matures. Expert Opin. Drug Deliv..

[67] Shi Y. (2020). Clinical Translation of Nanomedicine and Biomaterials for Cancer Immunotherapy: Progress and Perspectives. Adv. Ther..

[68] Younis M.A., Tawfeek H.M., Abdellatif A.A.H., Abdel-Aleem J.A., Harashima H. (2022). Clinical translation of nanomedicines: Challenges, opportunities, and keys. Adv. Drug Deliv. Rev..

[69] Zhang C.Y., Yan L., Wang X., Zhu S., Chen C.Y., Gu Z.J., Zhao Y.L. (2020). Progress, challenges, and future of nanomedicine. Nano Today.

[70] Zheng X., Xie J., Zhang X., Sun W., Zhao H., Li Y., Wang C. (2021). An overview of polymeric nanomicelles in clinical trials and on the market. Chin. Chem. Lett..

[71] Wang S., Cheng K., Chen K., Xu C., Ma P., Dang G., Yang Y., Lei Q., Huang H., Yu Y. (2022). Nanoparticle-based medicines in clinical cancer therapy. Nano Today.

[72] Rodriguez F., Caruana P., De la Fuente N., Espanol P., Gamez M., Balart J., Llurba E., Rovira R., Ruiz R., Martin-Lorente C. (2022). Nano-Based Approved Pharmaceuticals for Cancer Treatment: Present and Future Challenges. Biomolecules.

[73] Bagley A.F., Ludmir E.B., Maitra A., Minsky B.D., Li Smith G., Das P., Koong A.C., Holliday E.B., Taniguchi C.M., Katz M.H.G. (2022). NBTXR3, a first-in-class radioenhancer for pancreatic ductal adenocarcinoma: Report of first patient experience. Clin. Transl. Radiat. Oncol..

[74] Gong N., Sheppard N.C., Billingsley M.M., June C.H., Mitchell M.J. (2021). Nanomaterials for T-cell cancer immunotherapy. Nat. Nanotechnol..

[75] Li Y., Zhang X., Liu X., Pan W., Li N., Tang B. (2021). Intelligent stimuli-responsive nano immunomodulators for cancer immunotherapy. Chem. Sci..

[76] Li J., Lu W., Yang Y., Xiang R., Ling Y., Yu C., Zhou Y. (2023). Hybrid Nanomaterials for Cancer Immunotherapy. Adv. Sci..

[77] Thakur N., Thakur S., Chatterjee S., Das J., Sil P.C. (2020). Nanoparticles as Smart Carriers for Enhanced Cancer Immunotherapy. Front. Chem..

[78] Baeza A. (2020). Tumor Targeted Nanocarriers for Immunotherapy. Molecules.

[79] Rana I., Oh J., Baig J., Moon J.H., Son S., Nam J. (2023). Nanocarriers for cancer nano-immunotherapy. Drug Deliv. Transl. Res..

[80] Lee D., Huntoon K., Lux J., Kim B.Y.S., Jiang W. (2023). Engineering nanomaterial physical characteristics for cancer immunotherapy. Nat. Rev. Bioeng..

[81] Song W., Musetti S.N., Huang L. (2017). Nanomaterials for cancer immunotherapy. Biomaterials.

[82] Cabral H., Kinoh H., Kataoka K. (2020). Tumor-Targeted Nanomedicine for Immunotherapy. Acc. Chem. Res..

[83] Yu H.J., De Geest B.G. (2020). Nanomedicine and cancer immunotherapy. Acta Pharmacol. Sin..

[84] Bockamp E., Rosigkeit S., Siegl D., Schuppan D. (2020). Nano-Enhanced Cancer Immunotherapy: Immunology Encounters Nanotechnology. Cells.

[85] Nam J., Son S., Park K.S., Zou W., Shea L.D., Moon J.J. (2019). Cancer nanomedicine for combination cancer immunotherapy. Nat. Rev. Mater..

[86] Zhou L., Zou M., Xu Y., Lin P., Lei C., Xia X. (2022). Nano Drug Delivery System for Tumor Immunotherapy: Next-Generation Therapeutics. Front. Oncol..

[87] Yin W.M., Li Y.W., Gu Y.Q., Luo M. (2020). Nanoengineered targeting strategy for cancer immunotherapy. Acta Pharmacol. Sin..

[88] Ma J., Qiu J., Wang S. (2020). Nanozymes for Catalytic Cancer Immunotherapy. ACS Appl. Nano Mater..

[89] Irvine D.J., Dane E.L. (2020). Enhancing cancer immunotherapy with nanomedicine. Nat. Rev. Immunol..

[90] Zhao Z., Zheng L., Chen W., Weng W., Song J., Ji J. (2019). Delivery strategies of cancer immunotherapy: Recent advances and future perspectives. J. Hematol. Oncol..

[91] Yang J., Wang C., Shi S., Dong C. (2020). Nanotechnologies for enhancing cancer immunotherapy. Nano Res..

[92] Matsumura Y., Maeda H. (1986). A new concept for macromolecular therapeutics in cancer chemotherapy: Mechanism of tumoritropic accumulation of proteins and the antitumor agent smancs. Cancer Res..

[93] Ding Y.X., Xu Y.J., Yang W.Z., Niu P., Li X., Chen Y.D., Li Z.Y., Liu Y., An Y.L., Liu Y. (2020). Investigating the EPR effect of nanomedicines in human renal tumors perfusion strategy. Nano Today.

[94] Fang J., Islam W., Maeda H. (2020). Exploiting the dynamics of the EPR effect and strategies to improve the therapeutic effects of nanomedicines by using EPR effect enhancers. Adv. Drug Deliv. Rev..

[95] Shi Y., van der Meel R., Chen X., Lammers T. (2020). The EPR effect and beyond: Strategies to improve tumor targeting and cancer nanomedicine treatment efficacy. Theranostics.

[96] Prabhakar U., Maeda H., Jain R.K., Sevick-Muraca E.M., Zamboni W., Farokhzad O.C., Barry S.T., Gabizon A., Grodzinski P., Blakey D.C. (2013). Challenges and key considerations of the enhanced permeability and retention effect for nanomedicine drug delivery in oncology. Cancer Res..

[97] Xu W., Yang S., Lu L., Xu Q., Wu S., Zhou J., Lu J., Fan X., Meng N., Ding Y. (2023). Influence of lung cancer model characteristics on tumor targeting behavior of nanodrugs. J. Control. Release.

[98] Sun R., Xiang J., Zhou Q., Piao Y., Tang J., Shao S., Zhou Z., Bae Y.H., Shen Y. (2022). The tumor EPR effect for cancer drug delivery: Current status, limitations, and alternatives. Adv. Drug Deliv. Rev..

[99] Wilhelm S., Tavares A.J., Dai Q., Ohta S., Audet J., Dvorak H.F., Chan W.C.W. (2016). Analysis of nanoparticle delivery to tumours. Nat. Rev. Mater..

[100] Peng C., Huang Y., Zheng J. (2020). Renal clearable nanocarriers: Overcoming the physiological barriers for precise drug delivery and clearance. J. Control. Release.

[101] Zhang M., Ma H., Wang X., Yu B., Cong H., Shen Y. (2023). Polysaccharide-based nanocarriers for efficient transvascular drug delivery. J. Control. Release.

[102] Haynes M.T., Huang L. (2016). Multistage Delivery Technologies: Multifunctional, Interdisciplinary Approaches to Nanomedicine. Mol. Ther..

[103] Chen B., Dai W., He B., Zhang H., Wang X., Wang Y., Zhang Q. (2017). Current Multistage Drug Delivery Systems Based on the Tumor Microenvironment. Theranostics.

[104] Li M.M., Yu B., Wang S.C., Zhou F.J., Cui J., Su J.C. (2023). Microenvironment-responsive nanocarriers for targeted bone disease therapy. Nano Today.

[105] Li J., Gao X., Wang Y., Xia T., Zhao Y., Meng H. (2022). Precision design of engineered nanomaterials to guide immune systems for disease treatment. Matter.

[106] Shirsat S.D., Mane R.S., Thorat N.D., Kumar N. (2021). Nano-pharmacokinetics: Interface of physics, chemistry and biology. Nano-Pharmacokinetics and Theranostics.

[107] Xiao Y., Tang Z., Wang J., Liu C., Kong N., Farokhzad O.C., Tao W. (2020). Oral Insulin Delivery Platforms: Strategies To Address the Biological Barriers. Angew. Chem. Int. Ed..

[108] Kataoka K., Harada A., Nagasaki Y. (2012). Block copolymer micelles for drug delivery: Design, characterization and biological significance. Adv. Drug Deliv. Rev..

[109] Elsabahy M., Wooley K.L. (2012). Design of polymeric nanoparticles for biomedical delivery applications. Chem. Soc. Rev..

[110] Sun Q., Zhou Z., Qiu N., Shen Y. (2017). Rational Design of Cancer Nanomedicine: Nanoproperty Integration and Synchronization. Adv. Mater..

[111] Jing Wang Y.L. (2019). Guangjun Nie, Yuliang Zhao, recise design of nanomedicines: Perspectives for cancer treatment. Natl. Sci. Rev..

[112] Kobayashi H., Watanabe R., Choyke P.L. (2013). Improving conventional enhanced permeability and retention (EPR) effects; what is the appropriate target?. Theranostics.

[113] Park S.-m., Aalipour A., Vermesh O., Yu J.H., Gambhir S.S. (2017). Towards clinically translatable in vivo nanodiagnostics. Nat. Rev. Mater..

[114] Bourquin J., Milosevic A., Hauser D., Lehner R., Blank F., Petri-Fink A., Rothen-Rutishauser B. (2018). Biodistribution, Clearance, and Long-Term Fate of Clinically Relevant Nanomaterials. Adv. Mater..

[115] Mohsen K., Azzazy H.M.E., Allam N.K., Basalious E.B. (2020). Intranasal lipid nanocapsules for systemic delivery of nimodipine into the brain: In vitro optimization and in vivo pharmacokinetic study. Mater. Sci. Eng. C.

[116] Abtahi N.A., Salehi S., Naghib S.M., Haghiralsadat F., Edgahi M.A., Ghorbanzadeh S., Zhang W. (2023). Multi-sensitive functionalized niosomal nanocarriers for controllable gene delivery in vitro and in vivo. Cancer Nanotechnol..

[117] Yang M., Meng J., Han L., Yu X., Fan Z., Yuan Y. (2023). Pharmacokinetic Study of Triptolide Nanocarrier in Transdermal Drug Delivery System-Combination of Experiment and Mathematical Modeling. Molecules.

[118] Ouyang B., Poon W., Zhang Y.N., Lin Z.P., Kingston B.R., Tavares A.J., Zhang Y., Chen J., Valic M.S., Syed A.M. (2020). The dose threshold for nanoparticle tumour delivery. Nat. Mater..

[119] Meng H., Leong W., Leong K.W., Chen C., Zhao Y. (2018). Walking the line: The fate of nanomaterials at biological barriers. Biomaterials.

[120] Li Z., Xiao C., Yong T., Li Z., Gan L., Yang X. (2020). Influence of nanomedicine mechanical properties on tumor targeting delivery. Chem. Soc. Rev..

[121] Blanco E., Shen H., Ferrari M. (2015). Principles of nanoparticle design for overcoming biological barriers to drug delivery. Nat. Biotechnol..

[122] Dai Q., Wilhelm S., Ding D., Syed A.M., Sindhwani S., Zhang Y., Chen Y.Y., MacMillan P., Chan W.C.W. (2018). Quantifying the Ligand-Coated Nanoparticle Delivery to Cancer Cells in Solid Tumors. ACS Nano.

[123] Nienhaus K., Nienhaus G.U. (2023). Mechanistic Understanding of Protein Corona Formation around Nanoparticles: Old Puzzles and New Insights. Small.

[124] Ren J., Andrikopoulos N., Velonia K., Tang H., Cai R., Ding F., Ke P.C., Chen C. (2022). Chemical and Biophysical Signatures of the Protein Corona in Nanomedicine. J. Am. Chem. Soc..

[125] Cai R., Chen C. (2019). The Crown and the Scepter: Roles of the Protein Corona in Nanomedicine. Adv. Mater..

[126] Cai R., Ren J., Guo M., Wei T., Liu Y., Xie C., Zhang P., Guo Z., Chetwynd A.J., Ke P.C. (2022). Dynamic intracellular exchange of nanomaterials’ protein corona perturbs proteostasis and remodels cell metabolism. Proc. Natl. Acad. Sci. USA.

[127] Mahmoudi M., Landry M.P., Moore A., Coreas R. (2023). The protein corona from nanomedicine to environmental science. Nat. Rev. Mater..

[128] Cox T.R. (2021). The matrix in cancer. Nat. Rev. Cancer.

[129] Anderson N.M., Simon M.C. (2020). The tumor microenvironment. Curr. Biol..

[130] Huang J., Zhang L., Wan D., Zhou L., Zheng S., Lin S., Qiao Y. (2021). Extracellular matrix and its therapeutic potential for cancer treatment. Signal Transduct. Target. Ther..

[131] Zalba S., Ten Hagen T.L.M., Burgui C., Garrido M.J. (2022). Stealth nanoparticles in oncology: Facing the PEG dilemma. J. Control. Release.

[132] Salvati A., Pitek A.S., Monopoli M.P., Prapainop K., Bombelli F.B., Hristov D.R., Kelly P.M., Aberg C., Mahon E., Dawson K.A. (2013). Transferrin-functionalized nanoparticles lose their targeting capabilities when a biomolecule corona adsorbs on the surface. Nat. Nanotechnol..

[133] Jiang Z., Chu Y., Zhan C. (2022). Protein corona: Challenges and opportunities for targeted delivery of nanomedicines. Expert Opin. Drug Deliv..

[134] Kaur P. (2023). Deep dive into a drug pump. Nat. Chem. Biol..

[135] Vasan N., Baselga J., Hyman D.M. (2019). A view on drug resistance in cancer. Nature.

[136] Chen S., Zhong Y., Fan W., Xiang J., Wang G., Zhou Q., Wang J., Geng Y., Sun R., Zhang Z. (2021). Enhanced tumour penetration and prolonged circulation in blood of polyzwitterion-drug conjugates with cell-membrane affinity. Nat. Biomed. Eng..

[137] Cabral H., Matsumoto Y., Mizuno K., Chen Q., Murakami M., Kimura M., Terada Y., Kano M.R., Miyazono K., Uesaka M. (2011). Accumulation of sub-100 nm polymeric micelles in poorly permeable tumours depends on size. Nat. Nanotechnol..

[138] Watanabe S., Hayashi K., Toh K., Kim H.J., Liu X., Chaya H., Fukushima S., Katsushima K., Kondo Y., Uchida S. (2019). In vivo rendezvous of small nucleic acid drugs with charge-matched block catiomers to target cancers. Nat. Commun..

[139] Li Y.K., Zhong D., Zhou C.A., Tu Z.X., Mao H.L., Yang J., Zhang H., Luo K., Gong Q.Y., Gu Z.W. (2021). Sub-50 nm Supramolecular Nanohybrids with Active Targeting Corona for Image-Guided Solid Tumor Treatment and Metastasis Inhibition. Adv. Funct. Mater..

[140] Xu J., Song M., Fang Z., Zheng L., Huang X., Liu K. (2023). Applications and challenges of ultra-small particle size nanoparticles in tumor therapy. J. Control. Release.

[141] Petersen G.H., Alzghari S.K., Chee W., Sankari S.S., La-Beck N.M. (2016). Meta-analysis of clinical and preclinical studies comparing the anticancer efficacy of liposomal versus conventional non-liposomal doxorubicin. J. Control. Release.

[142] Maeda H. (2015). Toward a full understanding of the EPR effect in primary and metastatic tumors as well as issues related to its heterogeneity. Adv. Drug Deliv. Rev..

[143] Danhier F. (2016). To exploit the tumor microenvironment: Since the EPR effect fails in the clinic, what is the future of nanomedicine?. J. Control. Release.

[144] Mitchell M.J., Jain R.K., Langer R. (2017). Engineering and physical sciences in oncology: Challenges and opportunities. Nat. Rev. Cancer.

[145] Subhan M.A., Yalamarty S.S.K., Filipczak N., Parveen F., Torchilin V.P. (2021). Recent Advances in Tumor Targeting via EPR Effect for Cancer Treatment. J. Pers. Med..

[146] Srinivasarao M., Galliford C.V., Low P.S. (2015). Principles in the design of ligand-targeted cancer therapeutics and imaging agents. Nat. Rev. Drug Discov..

[147] Yao V.J., D’Angelo S., Butler K.S., Theron C., Smith T.L., Marchio S., Gelovani J.G., Sidman R.L., Dobroff A.S., Brinker C.J. (2016). Ligand-targeted theranostic nanomedicines against cancer. J. Control. Release.

[148] Ulbrich K., Hola K., Subr V., Bakandritsos A., Tucek J., Zboril R. (2016). Targeted drug delivery with polymers and magnetic nanoparticles: Covalent and noncovalent approaches, release control, and clinical studies. Chem. Rev..

[149] Biffi S., Voltan R., Bortot B., Zauli G., Secchiero P. (2019). Actively targeted nanocarriers for drug delivery to cancer cells. Expert Opin. Drug Deliv..

[150] Tylawsky D.E., Kiguchi H., Vaynshteyn J., Gerwin J., Shah J., Islam T., Boyer J.A., Boue D.R., Snuderl M., Greenblatt M.B. (2023). P-selectin-targeted nanocarriers induce active crossing of the blood-brain barrier via caveolin-1-dependent transcytosis. Nat. Mater..

[151] Salahpour Anarjan F. (2019). Active targeting drug delivery nanocarriers: Ligands. Nano-Struct. Nano-Objects.

[152] Zhao Z., Ukidve A., Kim J., Mitragotri S. (2020). Targeting Strategies for Tissue-Specific Drug Delivery. Cell.

[153] Khan N., Ruchika, Dhritlahre R.K., Saneja A. (2022). Recent advances in dual-ligand targeted nanocarriers for cancer therapy. Drug Discov. Today.

[154] Low P.S., Henne W.A., Doorneweerd D.D. (2008). Discovery and development of folic-acid-based receptor targeting for imaging and therapy of cancer and inflammatory diseases. Acc. Chem. Res..

[155] Attia M.F., Anton N., Wallyn J., Omran Z., Vandamme T.F. (2019). An overview of active and passive targeting strategies to improve the nanocarriers efficiency to tumour sites. J. Pharm. Pharmacol..

[156] Scaranti M., Cojocaru E., Banerjee S., Banerji U. (2020). Exploiting the folate receptor alpha in oncology. Nat. Rev. Clin. Oncol..

[157] Patra M., Awuah S.G., Lippard S.J. (2016). Chemical Approach to Positional Isomers of Glucose-Platinum Conjugates Reveals Specific Cancer Targeting through Glucose-Transporter-Mediated Uptake in Vitro and in Vivo. J. Am. Chem. Soc..

[158] Suzuki K., Miura Y., Mochida Y., Miyazaki T., Toh K., Anraku Y., Melo V., Liu X., Ishii T., Nagano O. (2019). Glucose transporter 1-mediated vascular translocation of nanomedicines enhances accumulation and efficacy in solid tumors. J. Control. Release.

[159] Zhou Y., Zhu F., Liu Y., Zheng M., Wang Y., Zhang D., Anraku Y., Zou Y., Li J., Wu H. (2020). Blood-brain barrier-penetrating siRNA nanomedicine for Alzheimer’s disease therapy. Sci. Adv..

[160] Su L., Feng Y., Wei K., Xu X., Liu R., Chen G. (2021). Carbohydrate-Based Macromolecular Biomaterials. Chem. Rev..

[161] Jain A., Jain A., Parajuli P., Mishra V., Ghoshal G., Singh B., Shivhare U.S., Katare O.P., Kesharwani P. (2018). Recent advances in galactose-engineered nanocarriers for the site-specific delivery of siRNA and anticancer drugs. Drug Discov. Today.

[162] Zuckerman J.E., Davis M.E. (2015). Clinical experiences with systemically administered siRNA-based therapeutics in cancer. Nat. Rev. Drug Discov..

[163] Johnston M.C., Scott C.J. (2018). Antibody conjugated nanoparticles as a novel form of antibody drug conjugate chemotherapy. Drug Discov. Today Technol..

[164] Paunovska K., Loughrey D., Dahlman J.E. (2022). Drug delivery systems for RNA therapeutics. Nat. Rev. Genet..

[165] Wu S.Y., Wu F.G., Chen X. (2022). Antibody-Incorporated Nanomedicines for Cancer Therapy. Adv. Mater..

[166] Tietz O., Cortezon-Tamarit F., Chalk R., Able S., Vallis K.A. (2022). Tricyclic cell-penetrating peptides for efficient delivery of functional antibodies into cancer cells. Nat. Chem..

[167] Raucher D. (2019). Tumor targeting peptides: Novel therapeutic strategies in glioblastoma. Curr. Opin. Pharmacol..

[168] Xie S., Sun W., Fu T., Liu X., Chen P., Qiu L., Qu F., Tan W. (2023). Aptamer-Based Targeted Delivery of Functional Nucleic Acids. J. Am. Chem. Soc..

[169] Wang X., Zhang X.-J., Li Y., Zhang G.-R., Li J., Wang X.-Q., Tan W. (2022). Molecularly Engineered Aptamers Targeting Tumor Tissue and Cancer Cells for Efficient in Vivo Recognition and Enrichment. CCS Chem..

[170] Liu Y., Qian X., Ran C., Li L., Fu T., Su D., Xie S., Tan W. (2023). Aptamer-Based Targeted Protein Degradation. ACS Nano.

[171] Alshaer W., Hillaireau H., Fattal E. (2018). Aptamer-guided nanomedicines for anticancer drug delivery. Adv. Drug Deliv. Rev..

[172] Xuan W., Peng Y., Deng Z., Peng T., Kuai H., Li Y., He J., Jin C., Liu Y., Wang R. (2018). A basic insight into aptamer-drug conjugates (ApDCs). Biomaterials.

[173] Zhu G., Chen X. (2018). Aptamer-based targeted therapy. Adv. Drug Deliv. Rev..

[174] Anraku Y., Kuwahara H., Fukusato Y., Mizoguchi A., Ishii T., Nitta K., Matsumoto Y., Toh K., Miyata K., Uchida S. (2017). Glycaemic control boosts glucosylated nanocarrier crossing the BBB into the brain. Nat. Commun..

[175] Min H.S., Kim H.J., Naito M., Ogura S., Toh K., Hayashi K., Kim B.S., Fukushima S., Anraku Y., Miyata K. (2020). Systemic Brain Delivery of Antisense Oligonucleotides across the Blood-Brain Barrier with a Glucose-Coated Polymeric Nanocarrier. Angew. Chem. Int. Ed..

[176] Yang T., Mochida Y., Liu X., Zhou H., Xie J., Anraku Y., Kinoh H., Cabral H., Kataoka K. (2021). Conjugation of glucosylated polymer chains to checkpoint blockade antibodies augments their efficacy and specificity for glioblastoma. Nat. Biomed. Eng..

[177] Yi Y., Kim H.J., Zheng M., Mi P., Naito M., Kim B.S., Min H.S., Hayashi K., Perche F., Toh K. (2019). Glucose-linked sub-50-nm unimer polyion complex-assembled gold nanoparticles for targeted siRNA delivery to glucose transporter 1-overexpressing breast cancer stem-like cells. J. Control. Release.

[178] Kim H.J., Takemoto H., Yi Y., Zheng M., Maeda Y., Chaya H., Hayashi K., Mi P., Pittella F., Christie R.J. (2014). Precise engineering of siRNA delivery vehicles to tumors using polyion complexes and gold nanoparticles. ACS Nano.

[179] Yi Y., Kim H.J., Mi P., Zheng M., Takemoto H., Toh K., Kim B.S., Hayashi K., Naito M., Matsumoto Y. (2016). Targeted systemic delivery of siRNA to cervical cancer model using cyclic RGD-installed unimer polyion complex-assembled gold nanoparticles. J. Control. Release.

[180] Khongorzul P., Ling C.J., Khan F.U., Ihsan A.U., Zhang J. (2020). Antibody-Drug Conjugates: A Comprehensive Review. Mol. Cancer Res..

[181] Drago J.Z., Modi S., Chandarlapaty S. (2021). Unlocking the potential of antibody-drug conjugates for cancer therapy. Nat. Rev. Clin. Oncol..

[182] Jabbour E., Paul S., Kantarjian H. (2021). The clinical development of antibody-drug conjugates-lessons from leukaemia. Nat. Rev. Clin. Oncol..

[183] Tarantino P., Ricciuti B., Pradhan S.M., Tolaney S.M. (2023). Optimizing the safety of antibody-drug conjugates for patients with solid tumours. Nat. Rev. Clin. Oncol..

[184] Dumontet C., Reichert J.M., Senter P.D., Lambert J.M., Beck A. (2023). Antibody-drug conjugates come of age in oncology. Nat. Rev. Drug Discov..

[185] Autio K.A., Dreicer R., Anderson J., Garcia J.A., Alva A., Hart L.L., Milowsky M.I., Posadas E.M., Ryan C.J., Graf R.P. (2018). Safety and Efficacy of BIND-014, a Docetaxel Nanoparticle Targeting Prostate-Specific Membrane Antigen for Patients With Metastatic Castration-Resistant Prostate Cancer: A Phase 2 Clinical Trial. JAMA Oncol..

[186] Ding Y., Wang Y., Hu Q. (2022). Recent advances in overcoming barriers to cell-based delivery systems for cancer immunotherapy. Exploration.

[187] Chanmee T., Ontong P., Konno K., Itano N. (2014). Tumor-associated macrophages as major players in the tumor microenvironment. Cancers.

[188] Giustarini G., Pavesi A., Adriani G. (2021). Nanoparticle-Based Therapies for Turning Cold Tumors Hot: How to Treat an Immunosuppressive Tumor Microenvironment. Front. Bioeng. Biotechnol..

[189] Zhang J., Huang D., Saw P.E., Song E. (2022). Turning cold tumors hot: From molecular mechanisms to clinical applications. Trends Immunol..

[190] Liu Y.T., Sun Z.J. (2021). Turning cold tumors into hot tumors by improving T-cell infiltration. Theranostics.

[191] Shi Y., Lammers T. (2019). Combining Nanomedicine and Immunotherapy. Acc. Chem. Res..

[192] Lakshmanan V.K., Jindal S., Packirisamy G., Ojha S., Lian S., Kaushik A., Alzarooni A., Metwally Y.A.F., Thyagarajan S.P., Do Jung Y. (2021). Nanomedicine-based cancer immunotherapy: Recent trends and future perspectives. Cancer Gene Ther..

[193] Chao Y., Liu Z. (2023). Biomaterials tools to modulate the tumour microenvironment in immunotherapy. Nat. Rev. Bioeng..

[194] Cheng R., Santos H.A. (2023). Smart Nanoparticle-Based Platforms for Regulating Tumor Microenvironment and Cancer Immunotherapy. Adv. Healthc. Mater..

[195] Mura S., Nicolas J., Couvreur P. (2013). Stimuli-responsive nanocarriers for drug delivery. Nat. Mater..

[196] Ge Z., Liu S. (2013). Functional block copolymer assemblies responsive to tumor and intracellular microenvironments for site-specific drug delivery and enhanced imaging performance. Chem. Soc. Rev..

[197] Torchilin V.P. (2014). Multifunctional, stimuli-sensitive nanoparticulate systems for drug delivery. Nat. Rev. Drug Discov..

[198] Xue X., Qu H., Li Y. (2022). Stimuli-responsive crosslinked nanomedicine for cancer treatment. Exploration.

[199] Zhang P., Gao D., An K., Shen Q., Wang C., Zhang Y., Pan X., Chen X., Lyv Y., Cui C. (2020). A programmable polymer library that enables the construction of stimuli-responsive nanocarriers containing logic gates. Nat. Chem..

[200] Mi P. (2020). Stimuli-responsive nanocarriers for drug delivery, tumor imaging, therapy and theranostics. Theranostics.

[201] Wang H., Wu H., Yi Y., Xue K.-F., Xu J.-F., Wang H., Zhao Y., Zhang X. (2021). Self-Motivated Supramolecular Combination Chemotherapy for Overcoming Drug Resistance Based on Acid-Activated Competition of Host–Guest Interactions. CCS Chem..

[202] Cong Y., Ji L., Gao Y.J., Liu F.H., Cheng D.B., Hu Z., Qiao Z.Y., Wang H. (2019). Microenvironment-Induced In Situ Self-Assembly of Polymer-Peptide Conjugates That Attack Solid Tumors Deeply. Angew. Chem. Int. Ed..

[203] Yang C., Wu X., Liu J., Ding B. (2022). Stimuli-responsive nucleic acid nanostructures for efficient drug delivery. Nanoscale.

[204] Liu M., Huang L., Zhang W., Wang X., Geng Y., Zhang Y., Wang L., Zhang W., Zhang Y.J., Xiao S. (2022). A transistor-like pH-sensitive nanodetergent for selective cancer therapy. Nat. Nanotechnol..

[205] Xu W., Luo F.-Q., Tong Q.-S., Li J.-X., Miao W.-M., Zhang Y., Xu C.-F., Du J.-Z., Wang J. (2021). An Intracellular pH-Actuated Polymer for Robust Cytosolic Protein Delivery. CCS Chem..

[206] Gong N., Zhang Y., Teng X., Wang Y., Huo S., Qing G., Ni Q., Li X., Wang J., Ye X. (2020). Proton-driven transformable nanovaccine for cancer immunotherapy. Nat. Nanotechnol..

[207] Chen Q., Wang C., Zhang X., Chen G., Hu Q., Li H., Wang J., Wen D., Zhang Y., Lu Y. (2019). In situ sprayed bioresponsive immunotherapeutic gel for post-surgical cancer treatment. Nat. Nanotechnol..

[208] Mi P., Kokuryo D., Cabral H., Wu H., Terada Y., Saga T., Aoki I., Nishiyama N., Kataoka K. (2016). A pH-activatable nanoparticle with signal-amplification capabilities for non-invasive imaging of tumour malignancy. Nat. Nanotechnol..

[209] Abed H.F., Abuwatfa W.H., Husseini G.A. (2022). Redox-Responsive Drug Delivery Systems: A Chemical Perspective. Nanomaterials.

[210] Cheng D.B., Zhang X.H., Gao Y.J., Ji L., Hou D., Wang Z., Xu W., Qiao Z.Y., Wang H. (2019). Endogenous Reactive Oxygen Species-Triggered Morphology Transformation for Enhanced Cooperative Interaction with Mitochondria. J. Am. Chem. Soc..

[211] Deng Z., Liu S. (2020). Controlled drug delivery with nanoassemblies of redox-responsive prodrug and polyprodrug amphiphiles. J. Control. Release.

[212] Mollazadeh S., Mackiewicz M., Yazdimamaghani M. (2021). Recent advances in the redox-responsive drug delivery nanoplatforms: A chemical structure and physical property perspective. Mater. Sci. Eng. C.

[213] Zheng M., Liu Y., Wang Y., Zhang D., Zou Y., Ruan W., Yin J., Tao W., Park J.B., Shi B. (2019). ROS-Responsive Polymeric siRNA Nanomedicine Stabilized by Triple Interactions for the Robust Glioblastoma Combinational RNAi Therapy. Adv. Mater..

[214] Liu T., Li L., Wang S., Dong F., Zuo S., Song J., Wang X., Lu Q., Wang H., Zhang H. (2022). Hybrid chalcogen bonds in prodrug nanoassemblies provides dual redox-responsivity in the tumor microenvironment. Nat. Commun..

[215] Cao Z., Li D., Wang J., Yang X. (2021). Reactive oxygen species-sensitive polymeric nanocarriers for synergistic cancer therapy. Acta Biomater..

[216] Pei P., Sun C., Tao W., Li J., Yang X., Wang J. (2019). ROS-sensitive thioketal-linked polyphosphoester-doxorubicin conjugate for precise phototriggered locoregional chemotherapy. Biomaterials.

[217] Liu X., Li M., Liu J., Song Y., Hu B., Wu C., Liu A.A., Zhou H., Long J., Shi L. (2022). In Situ Self-Sorting Peptide Assemblies in Living Cells for Simultaneous Organelle Targeting. J. Am. Chem. Soc..

[218] Wu H., Xia T., Qi F., Mei S., Xia Y., Xu J.-F., Zhang X. (2023). A Cleavable Self-Inclusion Conjugate with Enhanced Biocompatibility and Antitumor Bioactivity. CCS Chem..

[219] Li L., Zou J., Dai Y., Fan W., Niu G., Yang Z., Chen X. (2020). Burst release of encapsulated annexin A5 in tumours boosts cytotoxic T-cell responses by blocking the phagocytosis of apoptotic cells. Nat. Biomed. Eng..

[220] Yang J., Pan S., Gao S., Li T., Xu H. (2021). CO/chemosensitization/antiangiogenesis synergistic therapy with H2O2-responsive diselenide-containing polymer. Biomaterials.

[221] Dai Y., Li T., Zhang Z., Tan Y., Pan S., Zhang L., Xu H. (2021). Oxidative Polymerization in Living Cells. J. Am. Chem. Soc..

[222] Pan S., Yang J., Ji S., Li T., Gao S., Sun C., Xu H. (2020). Cancer Therapy by Targeting Thioredoxin Reductase Based on Selenium-Containing Dynamic Covalent Bond. CCS Chem..

[223] Li T., Xu H. (2020). Selenium-Containing Nanomaterials for Cancer Treatment. Cell Rep. Phys. Sci..

[224] Fan Z., Xu H. (2019). Recent Progress in the Biological Applications of Reactive Oxygen Species-Responsive Polymers. Polym. Rev..

[225] Ge C., Zhu J., Wu G., Ye H., Lu H., Yin L. (2022). ROS-Responsive Selenopolypeptide Micelles: Preparation, Characterization, and Controlled Drug Release. Biomacromolecules.

[226] Shi G., Cui Y., Zhao J., Liu J., Wang Y., Yang Y., Han J., Cheng X., Chen L., Yuan Y. (2023). Identifying TOPK and Hypoxia Hallmarks in Esophageal Tumors for Photodynamic/Chemo/Immunotherapy and Liver Metastasis Inhibition with Nanocarriers. ACS Nano.

[227] Liu J., Cabral H., Song B., Aoki I., Chen Z., Nishiyama N., Huang Y., Kataoka K., Mi P. (2021). Nanoprobe-Based Magnetic Resonance Imaging of Hypoxia Predicts Responses to Radiotherapy, Immunotherapy, and Sensitizing Treatments in Pancreatic Tumors. ACS Nano.

[228] Wang H., Xue K.F., Yang Y., Hu H., Xu J.F., Zhang X. (2022). In Situ Hypoxia-Induced Supramolecular Perylene Diimide Radical Anions in Tumors for Photothermal Therapy with Improved Specificity. J. Am. Chem. Soc..

[229] Chen H., Guo Q., Chu Y., Li C., Zhang Y., Liu P., Zhao Z., Wang Y., Luo Y., Zhou Z. (2022). Smart hypoxia-responsive transformable and charge-reversible nanoparticles for the deep penetration and tumor microenvironment modulation of pancreatic cancer. Biomaterials.

[230] Yuan R., Liu M., Cheng Y., Yan F., Zhu X., Zhou S., Dong M. (2023). Biomimetic Nanoparticle-Mediated Target Delivery of Hypoxia-Responsive Plasmid of Angiotensin-Converting Enzyme 2 to Reverse Hypoxic Pulmonary Hypertension. ACS Nano.

[231] Ge L., Qiao C., Tang Y., Zhang X., Jiang X. (2021). Light-Activated Hypoxia-Sensitive Covalent Organic Framework for Tandem-Responsive Drug Delivery. Nano Lett..

[232] He H., Lin X., Guo J., Wang J., Xu B. (2020). Perimitochondrial Enzymatic Self-Assembly for Selective Targeting the Mitochondria of Cancer Cells. ACS Nano.

[233] An H.W., Hou D., Zheng R., Wang M.D., Zeng X.Z., Xiao W.Y., Yan T.D., Wang J.Q., Zhao C.H., Cheng L.M. (2020). A Near-Infrared Peptide Probe with Tumor-Specific Excretion-Retarded Effect for Image-Guided Surgery of Renal Cell Carcinoma. ACS Nano.

[234] Ding Y., Zheng D., Xie L., Zhang X., Zhang Z., Wang L., Hu Z.W., Yang Z. (2023). Enzyme-Instructed Peptide Assembly Favored by Preorganization for Cancer Cell Membrane Engineering. J. Am. Chem. Soc..

[235] Gao J., Zhan J., Yang Z. (2020). Enzyme-Instructed Self-Assembly (EISA) and Hydrogelation of Peptides. Adv. Mater..

[236] Liu S., Zhang Q., Shy A.N., Yi M., He H., Lu S., Xu B. (2021). Enzymatically Forming Intranuclear Peptide Assemblies for Selectively Killing Human Induced Pluripotent Stem Cells. J. Am. Chem. Soc..

[237] He H., Guo J., Xu B. (2021). Enzymatic Delivery of Magnetic Nanoparticles into Mitochondria of Live Cells. ChemNanoMat.

[238] He H., Tan W., Guo J., Yi M., Shy A.N., Xu B. (2020). Enzymatic Noncovalent Synthesis. Chem. Rev..

[239] He H., Guo J., Lin X., Xu B. (2020). Enzyme-Instructed Assemblies Enable Mitochondria Localization of Histone H2B in Cancer Cells. Angew. Chem. Int. Ed..

[240] Wang Y.F., Kohane D.S. (2017). External triggering and triggered targeting strategies for drug delivery. Nat. Rev. Mater..

[241] Sun C., Gao S., Tan Y., Zhang Z., Xu H. (2021). Side-Chain Selenium-Grafted Polymers Combining Antiangiogenesis Treatment with Photodynamic Therapy and Chemotherapy. ACS Biomater. Sci. Eng..

[242] Zeng Z., Zhang C., Li J., Cui D., Jiang Y., Pu K. (2021). Activatable Polymer Nanoenzymes for Photodynamic Immunometabolic Cancer Therapy. Adv. Mater..

[243] Zhang X.H., Cheng D.B., Ji L., An H.W., Wang D., Yang Z.X., Chen H., Qiao Z.Y., Wang H. (2020). Photothermal-Promoted Morphology Transformation in Vivo Monitored by Photoacoustic Imaging. Nano Lett..

[244] Li L., Yang Z., Zhu S., He L., Fan W., Tang W., Zou J., Shen Z., Zhang M., Tang L. (2019). A Rationally Designed Semiconducting Polymer Brush for NIR-II Imaging-Guided Light-Triggered Remote Control of CRISPR/Cas9 Genome Editing. Adv. Mater..

[245] Wang X., Wu M., Li H., Jiang J., Zhou S., Chen W., Xie C., Zhen X., Jiang X. (2022). Enhancing Penetration Ability of Semiconducting Polymer Nanoparticles for Sonodynamic Therapy of Large Solid Tumor. Adv. Sci..

[246] Zhang H., Pan X., Wu Q., Guo J., Wang C., Liu H. (2021). Manganese carbonate nanoparticles-mediated mitochondrial dysfunction for enhanced sonodynamic therapy. Exploration.

[247] Fan J., Xuan M., Zhao P., Loznik M., Chen J., Kiessling F., Zheng L., Herrmann A. (2022). Ultrasound responsive microcapsules for antibacterial nanodrug delivery. Nano Res..

[248] Sun M., Yue T., Wang C., Fan Z., Gazit E., Du J. (2022). Ultrasound-Responsive Peptide Nanogels to Balance Conflicting Requirements for Deep Tumor Penetration and Prolonged Blood Circulation. ACS Nano.

[249] Du J.R., Wang Y., Yue Z.H., Zhang H.Y., Wang H., Sui G.Q., Sun Z.X. (2023). Recent advances in sonodynamic immunotherapy. J. Cancer Res. Clin. Oncol..

[250] Ho Y.J., Li J.P., Fan C.H., Liu H.L., Yeh C.K. (2020). Ultrasound in tumor immunotherapy: Current status and future developments. J. Control. Release.

[251] Ho W.J., Jaffee E.M., Zheng L. (2020). The tumour microenvironment in pancreatic cancer-clinical challenges and opportunities. Nat. Rev. Clin. Oncol..

[252] Tiwari A., Trivedi R., Lin S.Y. (2022). Tumor microenvironment: Barrier or opportunity towards effective cancer therapy. J. Biomed. Sci..

[253] Sherman M.H., Beatty G.L. (2023). Tumor Microenvironment in Pancreatic Cancer Pathogenesis and Therapeutic Resistance. Annu. Rev. Pathol..

[254] Arneth B. (2019). Tumor Microenvironment. Medicina.

[255] He Q., Chen J., Yan J., Cai S., Xiong H., Liu Y., Peng D., Mo M., Liu Z. (2020). Tumor microenvironment responsive drug delivery systems. Asian J. Pharm. Sci..

[256] He M., Wang M., Xu T., Zhang M., Dai H., Wang C., Ding D., Zhong Z. (2023). Reactive oxygen species-powered cancer immunotherapy: Current status and challenges. J. Control. Release.

[257] Jain R.K. (2013). Normalizing tumor microenvironment to treat cancer: Bench to bedside to biomarkers. J. Clin. Oncol..

[258] Zhao X., Amevor F.K., Xue X., Wang C., Cui Z., Dai S., Peng C., Li Y. (2023). Remodeling the hepatic fibrotic microenvironment with emerging nanotherapeutics: A comprehensive review. J. Nanobiotechnology.

[259] Liang C., Zhang X., Yang M., Wang W., Chen P., Dong X. (2020). Remodeling Tumor Microenvironment by Multifunctional Nanoassemblies for Enhanced Photodynamic Cancer Therapy. ACS Mater. Lett..

[260] Hauge A., Rofstad E.K. (2020). Antifibrotic therapy to normalize the tumor microenvironment. J. Transl. Med..

[261] Fukumura D., Kloepper J., Amoozgar Z., Duda D.G., Jain R.K. (2018). Enhancing cancer immunotherapy using antiangiogenics: Opportunities and challenges. Nat. Rev. Clin. Oncol..

[262] Li S.J., Chen J.X., Sun Z.J. (2021). Improving antitumor immunity using antiangiogenic agents: Mechanistic insights, current progress, and clinical challenges. Cancer Commun..

[263] Huinen Z.R., Huijbers E.J.M., van Beijnum J.R., Nowak-Sliwinska P., Griffioen A.W. (2021). Anti-angiogenic agents-overcoming tumour endothelial cell anergy and improving immunotherapy outcomes. Nat. Rev. Clin. Oncol..

[264] Dai Y., Xu C., Sun X., Chen X. (2017). Nanoparticle design strategies for enhanced anticancer therapy by exploiting the tumour microenvironment. Chem. Soc. Rev..

[265] Lin G., Chen S., Mi P. (2018). Nanoparticles Targeting and Remodeling Tumor Microenvironment for Cancer Theranostics. J. Biomed. Nanotechnol..

[266] Jin J., Ovais M., Chen C. (2018). Stimulus-responsive gold nanotheranostic platforms for targeting the tumor microenvironment. Nano Today.

[267] Lu H., Xu J., Yang J., Wang Z., Xu P., Hao Q., Luo W., Li S., Li Z., Xue X. (2022). On-demand targeting nanotheranostics with stimuli-responsive releasing property to improve delivery efficiency to cancer. Biomaterials.

[268] Liu H., Yao J., Guo H., Cai X., Jiang Y., Lin M., Jiang X., Leung W., Xu C. (2020). Tumor Microenvironment-Responsive Nanomaterials as Targeted Delivery Carriers for Photodynamic Anticancer Therapy. Front. Chem..

[269] Qin H., Ding Y., Mujeeb A., Zhao Y., Nie G. (2017). Tumor Microenvironment Targeting and Responsive Peptide-Based Nanoformulations for Improved Tumor Therapy. Mol. Pharmacol..

[270] Overchuk M., Zheng G. (2018). Overcoming obstacles in the tumor microenvironment: Recent advancements in nanoparticle delivery for cancer theranostics. Biomaterials.

[271] Zhou W., Jia Y., Liu Y., Chen Y., Zhao P. (2022). Tumor Microenvironment-Based Stimuli-Responsive Nanoparticles for Controlled Release of Drugs in Cancer Therapy. Pharmaceutics.

[272] Roma-Rodrigues C., Raposo L.R., Valente R., Fernandes A.R., Baptista P.V. (2021). Combined cancer therapeutics-Tackling the complexity of the tumor microenvironment. Wiley Interdiscip. Rev. Nanomed. Nanobiotechnol..

[273] Muntimadugu E., Kommineni N., Khan W. (2017). Exploring the Potential of Nanotherapeutics in Targeting Tumor Microenvironment for Cancer Therapy. Pharmacol. Res..

[274] Li J., Rao J., Pu K. (2018). Recent progress on semiconducting polymer nanoparticles for molecular imaging and cancer phototherapy. Biomaterials.

[275] Feng Q., Wilhelm J., Gao J. (2019). Transistor-like Ultra-pH-Sensitive Polymeric Nanoparticles. Acc. Chem. Res..

[276] Luo M., Wang H., Wang Z., Cai H., Lu Z., Li Y., Du M., Huang G., Wang C., Chen X. (2017). A STING-activating nanovaccine for cancer immunotherapy. Nat. Nanotechnol..

[277] Li S., Luo M., Wang Z., Feng Q., Wilhelm J., Wang X., Li W., Wang J., Cholka A., Fu Y.X. (2021). Prolonged activation of innate immune pathways by a polyvalent STING agonist. Nat. Biomed. Eng..

[278] Chen B., Yan Y., Yang Y., Cao G., Wang X., Wang Y., Wan F., Yin Q., Wang Z., Li Y. (2022). A pyroptosis nanotuner for cancer therapy. Nat. Nanotechnol..

[279] Dharmaratne N.U., Kaplan A.R., Glazer P.M. (2021). Targeting the Hypoxic and Acidic Tumor Microenvironment with pH-Sensitive Peptides. Cells.

[280] Wei X., Zhao H., Huang G., Liu J., He W., Huang Q. (2022). ES-MION-Based Dual-Modality PET/MRI Probes for Acidic Tumor Microenvironment Imaging. ACS Omega.

[281] Demin A.M., Pershina A.G., Minin A.S., Brikunova O.Y., Murzakaev A.M., Perekucha N.A., Romashchenko A.V., Shevelev O.B., Uimin M.A., Byzov I.V. (2021). Smart Design of a pH-Responsive System Based on pHLIP-Modified Magnetite Nanoparticles for Tumor MRI. ACS Appl. Mater. Interfaces.

[282] Ding G.B., Zhu C., Wang Q., Cao H., Li B.C., Yang P., Stauber R.H., Nie G., Li Z. (2022). Molecularly engineered tumor acidity-responsive plant toxin gelonin for safe and efficient cancer therapy. Bioact. Mater..

[283] Reshetnyak Y.K., Andreev O.A., Lehnert U., Engelman D.M. (2006). Translocation of molecules into cells by pH-dependent insertion of a transmembrane helix. Proc. Natl. Acad. Sci. USA.

[284] Chu T., Cao B., Wang P., Li B., Ren J., Nie G., Wei J., Li S. (2023). Tumor-Targeted Delivery of IL-2 by Fusing with a pH Low Insertion Peptide for Antitumor Immunotherapy. Bioconjug. Chem..

[285] Hu D., Zhang W., Xiang J., Li D., Chen Y., Yuan P., Shao S., Zhou Z., Shen Y., Tang J. (2022). A ROS-responsive synergistic delivery system for combined immunotherapy and chemotherapy. Mater. Today Bio..

[286] Xia H., Qin M., Wang Z., Wang Y., Chen B., Wan F., Tang M., Pan X., Yang Y., Liu J. (2022). A pH-/Enzyme-Responsive Nanoparticle Selectively Targets Endosomal Toll-like Receptors to Potentiate Robust Cancer Vaccination. Nano Lett..

[287] Li T., Zhang Y., Zhu J., Zhang F., Xu A., Zhou T., Li Y., Liu M., Ke H., Yang T. (2023). A pH-Activatable Copper-Biomineralized Proenzyme for Synergistic Chemodynamic/Chemo-Immunotherapy against Aggressive Cancers. Adv. Mater..

[288] Chang H.C., Zou Z.Z., Wang Q.H., Li J., Jin H., Yin Q.X., Xing D. (2020). Targeting and Specific Activation of Antigen-Presenting Cells by Endogenous Antigen-Loaded Nanoparticles Elicits Tumor-Specific Immunity. Adv. Sci..

[289] Li Y., Gong S., Pan W., Chen Y., Liu B., Li N., Tang B. (2020). A tumor acidity activatable and Ca(2+)-assisted immuno-nanoagent enhances breast cancer therapy and suppresses cancer recurrence. Chem. Sci..

[290] Zhou L., Hou B., Wang D., Sun F., Song R., Shao Q., Wang H., Yu H., Li Y. (2020). Engineering Polymeric Prodrug Nanoplatform for Vaccination Immunotherapy of Cancer. Nano Lett..

[291] Zhou P., Qin J., Zhou C., Wan G., Liu Y., Zhang M., Yang X., Zhang N., Wang Y. (2019). Multifunctional nanoparticles based on a polymeric copper chelator for combination treatment of metastatic breast cancer. Biomaterials.

[292] Wu J., Chen J., Feng Y., Zhang S., Lin L., Guo Z., Sun P., Xu C., Tian H., Chen X. (2020). An immune cocktail therapy to realize multiple boosting of the cancer-immunity cycle by combination of drug/gene delivery nanoparticles. Sci. Adv..

[293] Ji T., Lang J., Ning B., Qi F., Wang H., Zhang Y., Zhao R., Yang X., Zhang L., Li W. (2019). Enhanced Natural Killer Cell Immunotherapy by Rationally Assembling Fc Fragments of Antibodies onto Tumor Membranes. Adv. Mater..

[294] Chen Q., Chen G., Chen J., Shen J., Zhang X., Wang J., Chan A., Gu Z. (2019). Bioresponsive Protein Complex of aPD1 and aCD47 Antibodies for Enhanced Immunotherapy. Nano Lett..

[295] Yu S., Wang C., Yu J., Wang J., Lu Y., Zhang Y., Zhang X., Hu Q., Sun W., He C. (2018). Injectable Bioresponsive Gel Depot for Enhanced Immune Checkpoint Blockade. Adv. Mater..

[296] Ma S., Song W., Xu Y., Si X., Zhang Y., Tang Z., Chen X. (2020). A ROS-Responsive Aspirin Polymeric Prodrug for Modulation of Tumor Microenvironment and Cancer Immunotherapy. CCS Chem..

[297] Wan W.J., Qu C.X., Zhou Y.J., Zhang L., Chen M.T., Liu Y., You B.G., Li F., Wang D.D., Zhang X.N. (2019). Doxorubicin and siRNA-PD-L1 co-delivery with T7 modified ROS-sensitive nanoparticles for tumor chemoimmunotherapy. Int. J. Pharm..

[298] Pan S., Li T., Tan Y., Xu H. (2022). Selenium-containing nanoparticles synergistically enhance Pemetrexed&NK cell-based chemoimmunotherapy. Biomaterials.

[299] Liu Y., Lu Y., Zhu X., Li C., Yan M., Pan J., Ma G. (2020). Tumor microenvironment-responsive prodrug nanoplatform via co-self-assembly of photothermal agent and IDO inhibitor for enhanced tumor penetration and cancer immunotherapy. Biomaterials.

[300] Wen Y., Chen X., Zhu X., Gong Y., Yuan G., Qin X., Liu J. (2019). Photothermal-Chemotherapy Integrated Nanoparticles with Tumor Microenvironment Response Enhanced the Induction of Immunogenic Cell Death for Colorectal Cancer Efficient Treatment. ACS Appl. Mater. Interfaces.

[301] Li Q., Zhang D., Zhang J., Jiang Y., Song A., Li Z., Luan Y. (2019). A Three-in-One Immunotherapy Nanoweapon via Cascade-Amplifying Cancer-Immunity Cycle against Tumor Metastasis, Relapse, and Postsurgical Regrowth. Nano Lett..

[302] He Y., Lei L., Cao J., Yang X., Cai S., Tong F., Huang D., Mei H., Luo K., Gao H. (2021). A combinational chemo-immune therapy using an enzyme-sensitive nanoplatform for dual-drug delivery to specific sites by cascade targeting. Sci. Adv..

[303] Wang N., Zhou Y., Xu Y., Ren X., Zhou S., Shang Q., Jiang Y., Luan Y. (2020). Molecular engineering of anti-PD-L1 peptide and photosensitizer for immune checkpoint blockade photodynamic-immunotherapy. Chem. Eng. J..

[304] Gao A., Chen B., Gao J., Zhou F., Saeed M., Hou B., Li Y., Yu H. (2020). Sheddable Prodrug Vesicles Combating Adaptive Immune Resistance for Improved Photodynamic Immunotherapy of Cancer. Nano Lett..

[305] Im S., Lee J., Park D., Park A., Kim Y.M., Kim W.J. (2019). Hypoxia-Triggered Transforming Immunomodulator for Cancer Immunotherapy via Photodynamically Enhanced Antigen Presentation of Dendritic Cell. ACS Nano.

[306] Yang K., Yu G., Tian R., Zhou Z., Deng H., Li L., Yang Z., Zhang G., Liu D., Wei J. (2021). Oxygen-Evolving Manganese Ferrite Nanovesicles for Hypoxia-Responsive Drug Delivery and Enhanced Cancer Chemoimmunotherapy. Adv. Funct. Mater..

[307] Zhang L., Sun J., Huang W., Zhang S., Deng X., Gao W. (2023). Hypoxia-Triggered Bioreduction of Poly(N-oxide)-Drug Conjugates Enhances Tumor Penetration and Antitumor Efficacy. J. Am. Chem. Soc..

[308] Kang X., Zhang Y., Song J., Wang L., Li W., Qi J., Tang B.Z. (2023). A photo-triggered self-accelerated nanoplatform for multifunctional image-guided combination cancer immunotherapy. Nat. Commun..

[309] Zhang J., Li W., Qi Y., Wang G., Li L., Jin Z., Tian J., Du Y. (2023). PD-L1 Aptamer-Functionalized Metal-Organic Framework Nanoparticles for Robust Photo-Immunotherapy against Cancer with Enhanced Safety. Angew. Chem. Int. Ed..

[310] Qin H., Chen Y., Wang Z., Li N., Sun Q., Lin Y., Qiu W., Qin Y., Chen L., Chen H. (2023). Biosynthesized gold nanoparticles that activate Toll-like receptors and elicit localized light-converting hyperthermia for pleiotropic tumor immunoregulation. Nat. Commun..

[311] Zhu D.M., Chen H., Huang C.Y., Li G.X., Wang X., Jiang W., Fan K.L. (2022). H2O2 Self-Producing Single-Atom Nanozyme Hydrogels as Light-Controlled Oxidative Stress Amplifier for Enhanced Synergistic Therapy by Transforming “Cold” Tumors. Adv. Funct. Mater..

[312] Fu X., Huang Y., Zhao H., Zhang E., Shen Q., Di Y., Lv F., Liu L., Wang S. (2021). Near-Infrared-Light Remote-Controlled Activation of Cancer Immunotherapy Using Photothermal Conjugated Polymer Nanoparticles. Adv. Mater..

[313] Wang M., Song J., Zhou F., Hoover A.R., Murray C., Zhou B., Wang L., Qu J., Chen W.R. (2019). NIR-Triggered Phototherapy and Immunotherapy via an Antigen-Capturing Nanoplatform for Metastatic Cancer Treatment. Adv. Sci..

[314] Tan X., Huang J., Wang Y., He S., Jia L., Zhu Y., Pu K., Zhang Y., Yang X. (2021). Transformable Nanosensitizer with Tumor Microenvironment-Activated Sonodynamic Process and Calcium Release for Enhanced Cancer Immunotherapy. Angew. Chem. Int. Ed..

[315] Li J., Luo Y., Zeng Z., Cui D., Huang J., Xu C., Li L., Pu K., Zhang R. (2022). Precision cancer sono-immunotherapy using deep-tissue activatable semiconducting polymer immunomodulatory nanoparticles. Nat. Commun..

[316] Zhang C., Huang J., Zeng Z., He S., Cheng P., Li J., Pu K. (2022). Catalytical nano-immunocomplexes for remote-controlled sono-metabolic checkpoint trimodal cancer therapy. Nat. Commun..

[317] Meng Z., Zhang Y., She J., Zhou X., Xu J., Han X., Wang C., Zhu M., Liu Z. (2021). Ultrasound-Mediated Remotely Controlled Nanovaccine Delivery for Tumor Vaccination and Individualized Cancer Immunotherapy. Nano Lett..

[318] Abedi M.H., Yao M.S., Mittelstein D.R., Bar-Zion A., Swift M.B., Lee-Gosselin A., Barturen-Larrea P., Buss M.T., Shapiro M.G. (2022). Ultrasound-controllable engineered bacteria for cancer immunotherapy. Nat. Commun..

[319] Chen Y., Du M., Yuan Z., Chen Z., Yan F. (2022). Spatiotemporal control of engineered bacteria to express interferon-gamma by focused ultrasound for tumor immunotherapy. Nat. Commun..

[320] Shao D., Zhang F., Chen F., Zheng X., Hu H., Yang C., Tu Z., Wang Z., Chang Z., Lu J. (2020). Biomimetic Diselenide-Bridged Mesoporous Organosilica Nanoparticles as an X-ray-Responsive Biodegradable Carrier for Chemo-Immunotherapy. Adv. Mater..

[321] Li T., Pan S., Gao S., Xiang W., Sun C., Cao W., Xu H. (2020). Diselenide-Pemetrexed Assemblies for Combined Cancer Immuno-, Radio-, and Chemotherapies. Angew. Chem. Int. Ed..

[322] Gao S., Li T., Guo Y., Sun C., Xianyu B., Xu H. (2020). Selenium-Containing Nanoparticles Combine the NK Cells Mediated Immunotherapy with Radiotherapy and Chemotherapy. Adv. Mater..

[323] Chang Y., Huang J., Shi S., Xu L., Lin H., Chen T. (2023). Precise Engineering of a Se/Te Nanochaperone for Reinvigorating Cancer Radio-Immunotherapy. Adv. Mater..

[324] Yang G., Liu J., Wu Y., Feng L., Liu Z. (2016). Near-infrared-light responsive nanoscale drug delivery systems for cancer treatment. Coord. Chem. Rev..

[325] Li J.-B., Liu H.-W., Fu T., Wang R., Zhang X.-B., Tan W. (2019). Recent Progress in Small-Molecule Near-IR Probes for Bioimaging. Trends Chem..

[326] Zhu S., Yung B.C., Chandra S., Niu G., Antaris A.L., Chen X. (2018). Near-infrared-II (NIR-II) bioimaging via off-peak NIR-I fluorescence emission. Theranostics.

[327] Zhu S., Tian R., Antaris A.L., Chen X., Dai H. (2019). Near-Infrared-II Molecular Dyes for Cancer Imaging and Surgery. Adv. Mater..

[328] Jiang Y., Huang J., Xu C., Pu K. (2021). Activatable polymer nanoagonist for second near-infrared photothermal immunotherapy of cancer. Nat. Commun..

[329] Chen A., Wu L., Luo Y., Lu S., Wang Y., Zhou Z., Zhou D., Xie Z., Yue J. (2022). Deep Tumor Penetrating Gold Nano-Adjuvant for NIR-II-Triggered In Situ Tumor Vaccination. Small.

[330] Mitragotri S. (2005). Healing sound: The use of ultrasound in drug delivery and other therapeutic applications. Nat. Rev. Drug Discov..

[331] Boissenot T., Bordat A., Fattal E., Tsapis N. (2016). Ultrasound-triggered drug delivery for cancer treatment using drug delivery systems: From theoretical considerations to practical applications. J. Control. Release.

[332] Tu L., Liao Z., Luo Z., Wu Y.L., Herrmann A., Huo S. (2021). Ultrasound-controlled drug release and drug activation for cancer therapy. Exploration.

[333] Moradi Kashkooli F., Jakhmola A., Hornsby T.K., Tavakkoli J.J., Kolios M.C. (2023). Ultrasound-mediated nano drug delivery for treating cancer: Fundamental physics to future directions. J. Control. Release.

[334] Cui X.W., Han X.X., Yu L.D., Zhang B., Chen Y. (2019). Intrinsic chemistry and design principle of ultrasound-responsive nanomedicine. Nano Today.

[335] Entzian K., Aigner A. (2021). Drug Delivery by Ultrasound-Responsive Nanocarriers for Cancer Treatment. Pharmaceutics.

[336] Tak W.Y., Lin S.M., Wang Y., Zheng J., Vecchione A., Park S.Y., Chen M.H., Wong S., Xu R., Peng C.Y. (2018). Phase III HEAT Study Adding Lyso-Thermosensitive Liposomal Doxorubicin to Radiofrequency Ablation in Patients with Unresectable Hepatocellular Carcinoma Lesions. Clin. Cancer. Res..

[337] Li X., Khorsandi S., Wang Y., Santelli J., Huntoon K., Nguyen N., Yang M., Lee D., Lu Y., Gao R. (2022). Cancer immunotherapy based on image-guided STING activation by nucleotide nanocomplex-decorated ultrasound microbubbles. Nat. Nanotechnol..

[338] Jiang J., Zhang M., Lyu T., Chen L., Wu M., Li R., Li H., Wang X., Jiang X., Zhen X. (2023). Sono-Driven STING Activation using Semiconducting Polymeric Nanoagonists for Precision Sono-Immunotherapy of Head and Neck Squamous Cell Carcinoma. Adv. Mater..

[339] Zhang Q., Shi D., Guo M., Zhao H., Zhao Y., Yang X. (2023). Radiofrequency-Activated Pyroptosis of Bi-Valent Gold Nanocluster for Cancer Immunotherapy. ACS Nano.

[340] Chao C.J., Zhang E., Zhao Z. (2023). Engineering cells for precision drug delivery: New advances, clinical translation, and emerging strategies. Adv. Drug Deliv. Rev..

[341] Finbloom J.A., Sousa F., Stevens M.M., Desai T.A. (2020). Engineering the drug carrier biointerface to overcome biological barriers to drug delivery. Adv. Drug Deliv. Rev..

[342] Mitchell M.J., Billingsley M.M., Haley R.M., Wechsler M.E., Peppas N.A., Langer R. (2021). Engineering precision nanoparticles for drug delivery. Nat. Rev. Drug Discov..

[343] Tasciotti E., Liu X., Bhavane R., Plant K., Leonard A.D., Price B.K., Cheng M.M., Decuzzi P., Tour J.M., Robertson F. (2008). Mesoporous silicon particles as a multistage delivery system for imaging and therapeutic applications. Nat. Nanotechnol..

[344] Serda R.E., Godin B., Blanco E., Chiappini C., Ferrari M. (2011). Multi-stage delivery nano-particle systems for therapeutic applications. Biochim. Biophys. Acta.

[345] Godin B., Tasciotti E., Liu X., Serda R.E., Ferrari M. (2011). Multistage nanovectors: From concept to novel imaging contrast agents and therapeutics. Acc. Chem. Res..

[346] Martinez J.O., Brown B.S., Quattrocchi N., Evangelopoulos M., Ferrari M., Tasciotti E. (2012). Multifunctional to multistage delivery systems: The evolution of nanoparticles for biomedical applications. Chin. Sci. Bull..

[347] Mi Y., Wolfram J., Mu C., Liu X., Blanco E., Shen H., Ferrari M. (2016). Enzyme-responsive multistage vector for drug delivery to tumor tissue. Pharmacol. Res..

[348] Venuta A., Wolfram J., Shen H., Ferrari M. (2017). Post-nano strategies for drug delivery: Multistage porous silicon microvectors. J. Mater. Chem. B.

[349] Xu R., Zhang G., Mai J., Deng X., Segura-Ibarra V., Wu S., Shen J., Liu H., Hu Z., Chen L. (2016). An injectable nanoparticle generator enhances delivery of cancer therapeutics. Nat. Biotechnol..

[350] Kim J., Lee S., Kim Y., Choi M., Lee I., Kim E., Yoon C.G., Pu K., Kang H.M., Kim J.S. (2023). In situ self-assembly for cancer therapy and imaging. Nat. Rev. Mater..

[351] Chagri S., Ng D.Y.W., Weil T. (2022). Designing bioresponsive nanomaterials for intracellular self-assembly. Nat. Rev. Chem..

[352] Mamuti M., Zheng R., An H.W., Wang H. (2021). In vivo self-assembled nanomedicine. Nano Today.

[353] Feng Y., Xie X., Zhang H., Su Q., Yang G., Wei X., Li N., Li T., Qin X., Li S. (2021). Multistage-responsive nanovehicle to improve tumor penetration for dual-modality imaging-guided photodynamic-immunotherapy. Biomaterials.

[354] Wang J., Shen S., Li J., Cao Z., Yang X. (2021). Precise Depletion of Tumor Seed and Growing Soil with Shrinkable Nanocarrier for Potentiated Cancer Chemoimmunotherapy. ACS Nano.

[355] Zhang C., Zeng Z., Cui D., He S., Jiang Y., Li J., Huang J., Pu K. (2021). Semiconducting polymer nano-PROTACs for activatable photo-immunometabolic cancer therapy. Nat. Commun..

[356] Feng B., Hou B., Xu Z., Saeed M., Yu H., Li Y. (2019). Self-Amplified Drug Delivery with Light-Inducible Nanocargoes to Enhance Cancer Immunotherapy. Adv. Mater..

[357] Wang M.D., Lv G.T., An H.W., Zhang N.Y., Wang H. (2022). In Situ Self-Assembly of Bispecific Peptide for Cancer Immunotherapy. Angew. Chem. Int. Ed..

[358] Wang C., Wang J., Zhang X., Yu S., Wen D., Hu Q., Ye Y., Bomba H., Hu X., Liu Z. (2018). In situ formed reactive oxygen species-responsive scaffold with gemcitabine and checkpoint inhibitor for combination therapy. Sci. Transl. Med..

[359] Lv M.Y., Xiao W.Y., Zhang Y.P., Jin L.L., Li Z.H., Lei Z., Cheng D.B., Jin S.D. (2022). In situ self-assembled peptide enables effective cancer immunotherapy by blockage of CD47. Colloids Surf. B. Biointerfaces.

[360] Li X., Jeon Y.H., Kwon N., Park J.G., Guo T., Kim H.R., Huang J.D., Lee D.S., Yoon J. (2021). In Vivo-assembled phthalocyanine/albumin supramolecular complexes combined with a hypoxia-activated prodrug for enhanced photodynamic immunotherapy of cancer. Biomaterials.

[361] Sun L., Shen F., Xiong Z., Chao Y., Fan C., Liu Z. (2023). Nanoscale Precise Editing of Multiple Immune Stimulating Ligands on DNA Origami for T Cell Activation and Cell-Based Cancer Immunotherapy. CCS Chem..

[362] Sun L., Shen F., Xu J., Han X., Fan C., Liu Z. (2020). DNA-Edited Ligand Positioning on Red Blood Cells to Enable Optimized T Cell Activation for Adoptive Immunotherapy. Angew. Chem. Int. Ed..

[363] Sun L., Shen F., Xiong Z., Yang H., Dong Z., Xiang J., Gu Q., Ji Q., Fan C., Liu Z. (2022). DNA Engineered Lymphocyte-Based Homologous Targeting Artificial Antigen-Presenting Cells for Personalized Cancer Immunotherapy. J. Am. Chem. Soc..

[364] Xu C.F., Zhang H.B., Sun C.Y., Liu Y., Shen S., Yang X.Z., Zhu Y.H., Wang J. (2016). Tumor acidity-sensitive linkage-bridged block copolymer for therapeutic siRNA delivery. Biomaterials.

[365] Sun C.Y., Shen S., Xu C.F., Li H.J., Liu Y., Cao Z.T., Yang X.Z., Xia J.X., Wang J. (2015). Tumor acidity-sensitive polymeric vector for active targeted siRNA delivery. J. Am. Chem. Soc..

[366] Li X., Gao Y., Li H., Majoral J.-P., Shi X., Pich A. (2023). Smart and bioinspired systems for overcoming biological barriers and enhancing disease theranostics. Prog. Mater. Sci..

[367] Olson E.S., Jiang T., Aguilera T.A., Nguyen Q.T., Ellies L.G., Scadeng M., Tsien R.Y. (2010). Activatable cell penetrating peptides linked to nanoparticles as dual probes for in vivo fluorescence and MR imaging of proteases. Proc. Natl. Acad. Sci. USA.

[368] Ke W., Zha Z., Mukerabigwi J.F., Chen W., Wang Y., He C., Ge Z. (2017). Matrix Metalloproteinase-Responsive Multifunctional Peptide-Linked Amphiphilic Block Copolymers for Intelligent Systemic Anticancer Drug Delivery. Bioconjug. Chem..

[369] Xu X., Saw P.E., Tao W., Li Y., Ji X., Yu M., Mahmoudi M., Rasmussen J., Ayyash D., Zhou Y. (2017). Tumor Microenvironment-Responsive Multistaged Nanoplatform for Systemic RNAi and Cancer Therapy. Nano Lett..

[370] Li H.J., Du J.Z., Liu J., Du X.J., Shen S., Zhu Y.H., Wang X., Ye X., Nie S., Wang J. (2016). Smart Superstructures with Ultrahigh pH-Sensitivity for Targeting Acidic Tumor Microenvironment: Instantaneous Size Switching and Improved Tumor Penetration. ACS Nano.

[371] Li H.J., Du J.Z., Du X.J., Xu C.F., Sun C.Y., Wang H.X., Cao Z.T., Yang X.Z., Zhu Y.H., Nie S. (2016). Stimuli-responsive clustered nanoparticles for improved tumor penetration and therapeutic efficacy. Proc. Natl. Acad. Sci. USA.

[372] Tang L., Yang X., Yin Q., Cai K., Wang H., Chaudhury I., Yao C., Zhou Q., Kwon M., Hartman J.A. (2014). Investigating the optimal size of anticancer nanomedicine. Proc. Natl. Acad. Sci. USA.

[373] Wang J., Mao W., Lock L.L., Tang J., Sui M., Sun W., Cui H., Xu D., Shen Y. (2015). The role of micelle size in tumor accumulation, penetration, and treatment. ACS Nano.

[374] Li J., Ke W., Li H., Zha Z., Han Y., Ge Z. (2015). Endogenous stimuli-sensitive multistage polymeric micelleplex anticancer drug delivery system for efficient tumor penetration and cellular internalization. Adv. Healthc. Mater..

[375] Sun Q., Ojha T., Kiessling F., Lammers T., Shi Y. (2017). Enhancing Tumor Penetration of Nanomedicines. Biomacromolecules.

[376] Kim H.J., Yi Y., Kim A., Miyata K. (2018). Small Delivery Vehicles of siRNA for Enhanced Cancer Targeting. Biomacromolecules.

[377] Shu M., Tang J., Chen L., Zeng Q., Li C., Xiao S., Jiang Z., Liu J. (2021). Tumor microenvironment triple-responsive nanoparticles enable enhanced tumor penetration and synergetic chemo-photodynamic therapy. Biomaterials.

[378] Rautio J., Meanwell N.A., Di L., Hageman M.J. (2018). The expanding role of prodrugs in contemporary drug design and development. Nat. Rev. Drug Discov..

[379] Najjar A., Najjar A., Karaman R. (2020). Newly Developed Prodrugs and Prodrugs in Development; an Insight of the Recent Years. Molecules.

[380] Detappe A., Nguyen H.V., Jiang Y., Agius M.P., Wang W., Mathieu C., Su N.K., Kristufek S.L., Lundberg D.J., Bhagchandani S. (2023). Molecular bottlebrush prodrugs as mono- and triplex combination therapies for multiple myeloma. Nat. Nanotechnol..

[381] Ding C., Chen C., Zeng X., Chen H., Zhao Y. (2022). Emerging Strategies in Stimuli-Responsive Prodrug Nanosystems for Cancer Therapy. ACS Nano.

[382] Dong X., Brahma R.K., Fang C., Yao S.Q. (2022). Stimulus-responsive self-assembled prodrugs in cancer therapy. Chem. Sci..

[383] Zhang P., Zhang Y., Ding X., Shen W., Li M., Wagner E., Xiao C., Chen X. (2020). A Multistage Cooperative Nanoplatform Enables Intracellular Co-Delivery of Proteins and Chemotherapeutics for Cancer Therapy. Adv. Mater..

[384] Zhang Y., Ma S., Liu X., Xu Y., Zhao J., Si X., Li H., Huang Z., Wang Z., Tang Z. (2021). Supramolecular Assembled Programmable Nanomedicine As In Situ Cancer Vaccine for Cancer Immunotherapy. Adv. Mater..

[385] Feng L.D., Yang L., Li L.J., Xiao J.Y., Bie N.N., Xu C., Zhou J., Liu H.M., Gan L., Wu Y.Z. (2022). Programmed albumin nanoparticles regulate immunosuppressive pivot to potentiate checkpoint blockade cancer immunotherapy. Nano Res..

[386] Wei Z., Yi Y., Luo Z., Gong X., Jiang Y., Hou D., Zhang L., Liu Z., Wang M., Wang J. (2022). Selenopeptide Nanomedicine Activates Natural Killer Cells for Enhanced Tumor Chemoimmunotherapy. Adv. Mater..

[387] Bressler E.M., Adams S., Liu R., Colson Y.L., Wong W.W., Grinstaff M.W. (2023). Boolean logic in synthetic biology and biomaterials: Towards living materials in mammalian cell therapeutics. Clin. Transl. Med..

[388] Luo C., He L., Chen F., Fu T., Zhang P., Xiao Z., Liu Y., Tan W. (2021). Stimulus-responsive nanomaterials containing logic gates for biomedical applications. Cell Rep. Phys. Sci..

[389] Badeau B.A., Comerford M.P., Arakawa C.K., Shadish J.A., DeForest C.A. (2018). Engineered modular biomaterial logic gates for environmentally triggered therapeutic delivery. Nat. Chem..

[390] Mamuti M., Wang Y., Zhao Y.D., Wang J.Q., Wang J., Fan Y.L., Xiao W.Y., Hou D.Y., Yang J., Zheng R. (2022). A Polyvalent Peptide CD40 Nanoagonist for Targeted Modulation of Dendritic Cells and Amplified Cancer Immunotherapy. Adv. Mater..

[391] Wang M.D., Hou D.Y., Lv G.T., Li R.X., Hu X.J., Wang Z.J., Zhang N.Y., Yi L., Xu W.H., Wang H. (2021). Targeted in situ self-assembly augments peptide drug conjugate cell-entry efficiency. Biomaterials.

[392] Wang J., Yi W., Qiao S., Mamuti M., An H., Wang H. (2021). In situ phase transitional polymeric vaccines for improved immunotherapy. Natl. Sci. Rev..

[393] Wang Y., Li X., Zheng D., Chen Y., Zhang Z., Yang Z. (2021). Selective Degradation of PD-L1 in Cancer Cells by Enzyme-Instructed Self-Assembly. Adv. Funct. Mater..

[394] Li J., Fang Y., Zhang Y., Wang H., Yang Z., Ding D. (2021). Supramolecular Self-Assembly-Facilitated Aggregation of Tumor-Specific Transmembrane Receptors for Signaling Activation and Converting Immunologically Cold to Hot Tumors. Adv. Mater..

[395] Hu X.J., Zhang N.Y., Hou D.Y., Wang Z.J., Wang M.D., Yi L., Song Z.Z., Liang J.X., Li X.P., An H.W. (2023). An In Vivo Self-Assembled Bispecific Nanoblocker for Enhancing Tumor Immunotherapy. Adv. Mater..

[396] Larson R.C., Maus M.V. (2021). Recent advances and discoveries in the mechanisms and functions of CAR T cells. Nat. Rev. Cancer.

[397] Nahmad A.D., Lazzarotto C.R., Zelikson N., Kustin T., Tenuta M., Huang D., Reuveni I., Nataf D., Raviv Y., Horovitz-Fried M. (2022). In vivo engineered B cells secrete high titers of broadly neutralizing anti-HIV antibodies in mice. Nat. Biotechnol..

[398] Jiang C.T., Chen K.G., Liu A., Huang H., Fan Y.N., Zhao D.K., Ye Q.N., Zhang H.B., Xu C.F., Shen S. (2021). Immunomodulating nano-adaptors potentiate antibody-based cancer immunotherapy. Nat. Commun..

[399] Zhao Y.D., An H.W., Mamuti M., Zeng X.Z., Zheng R., Yang J., Zhou W., Liang Y., Qin G., Hou D.Y. (2023). Reprogramming Hypoxic Tumor-Associated Macrophages by Nanoglycoclusters for Boosted Cancer Immunotherapy. Adv. Mater..

[400] An H.W., Hou D.Y., Yang J., Wang Z.Q., Wang M.D., Zheng R., Zhang N.Y., Hu X.J., Wang Z.J., Wang L. (2023). A bispecific glycopeptide spatiotemporally regulates tumor microenvironment for inhibiting bladder cancer recurrence. Sci. Adv..

